# Natural Language Processing Reveals Vulnerable Mental Health Support Groups and Heightened Health Anxiety on Reddit During COVID-19: Observational Study

**DOI:** 10.2196/22635

**Published:** 2020-10-12

**Authors:** Daniel M Low, Laurie Rumker, Tanya Talkar, John Torous, Guillermo Cecchi, Satrajit S Ghosh

**Affiliations:** 1 Program in Speech and Hearing Bioscience and Technology Harvard Medical School Boston, MA United States; 2 Department of Brain and Cognitive Sciences Massachusetts Institute of Technology Cambridge, MA United States; 3 McGovern Institute for Brain Research Massachusetts Institute of Technology Cambridge, MA United States; 4 Center for Data Sciences Brigham and Women’s Hospital and Harvard Medical School Boston, MA United States; 5 Department of Medicine Brigham and Women’s Hospital and Harvard Medical School Boston, MA United States; 6 Broad Institute of Massachusetts Institute of Technology and Harvard Cambridge, MA United States; 7 Department of Biomedical Informatics Harvard Medical School Boston, MA United States; 8 Human Health and Performance Systems Massachusetts Institute of Technology Lincoln Laboratory Lexington, MA United States; 9 Digital Psychiatry Division Department of Psychiatry at Beth Israel Deaconess Medical Center Harvard Medical School Boston, MA United States; 10 Thomas J Watson Research Center IBM Yorktown Heights, NY United States; 11 Department of Otolaryngology Harvard Medical School Boston, MA United States

**Keywords:** COVID-19, mental health, psychiatry, infodemiology, infoveillance, infodemic, social media, Reddit, natural language processing, ADHD, eating disorders, anxiety, suicidality

## Abstract

**Background:**

The COVID-19 pandemic is impacting mental health, but it is not clear how people with different types of mental health problems were differentially impacted as the initial wave of cases hit.

**Objective:**

The aim of this study is to leverage natural language processing (NLP) with the goal of characterizing changes in 15 of the world’s largest mental health support groups (eg, r/schizophrenia, r/SuicideWatch, r/Depression) found on the website Reddit, along with 11 non–mental health groups (eg, r/PersonalFinance, r/conspiracy) during the initial stage of the pandemic.

**Methods:**

We created and released the Reddit Mental Health Dataset including posts from 826,961 unique users from 2018 to 2020. Using regression, we analyzed trends from 90 text-derived features such as sentiment analysis, personal pronouns, and semantic categories. Using supervised machine learning, we classified posts into their respective support groups and interpreted important features to understand how different problems manifest in language. We applied unsupervised methods such as topic modeling and unsupervised clustering to uncover concerns throughout Reddit before and during the pandemic.

**Results:**

We found that the r/HealthAnxiety forum showed spikes in posts about COVID-19 early on in January, approximately 2 months before other support groups started posting about the pandemic. There were many features that significantly increased during COVID-19 for specific groups including the categories “economic stress,” “isolation,” and “home,” while others such as “motion” significantly decreased. We found that support groups related to attention-deficit/hyperactivity disorder, eating disorders, and anxiety showed the most negative semantic change during the pandemic out of all mental health groups. Health anxiety emerged as a general theme across Reddit through independent supervised and unsupervised machine learning analyses. For instance, we provide evidence that the concerns of a diverse set of individuals are converging in this unique moment of history; we discovered that the more users posted about COVID-19, the more linguistically similar (less distant) the mental health support groups became to r/HealthAnxiety (ρ=–0.96, *P*<.001). Using unsupervised clustering, we found the suicidality and loneliness clusters more than doubled in the number of posts during the pandemic. Specifically, the support groups for borderline personality disorder and posttraumatic stress disorder became significantly associated with the suicidality cluster. Furthermore, clusters surrounding self-harm and entertainment emerged.

**Conclusions:**

By using a broad set of NLP techniques and analyzing a baseline of prepandemic posts, we uncovered patterns of how specific mental health problems manifest in language, identified at-risk users, and revealed the distribution of concerns across Reddit, which could help provide better resources to its millions of users. We then demonstrated that textual analysis is sensitive to uncover mental health complaints as they appear in real time, identifying vulnerable groups and alarming themes during COVID-19, and thus may have utility during the ongoing pandemic and other world-changing events such as elections and protests.

## Introduction

The ongoing outbreak of a novel coronavirus causing the disease COVID-19 is likely to have impacts on mental health as many individuals experience losses of income, social engagement, mobility, physical health, and uncertainty. Characterizing these impacts is critical to motivate and inform the provision of appropriate therapeutic responses. Public commentary posted to mental health support groups on the website Reddit through subfora (subreddits) captures in real time the language used by those sharing and processing their pandemic experiences online. In this study, we apply text processing and machine learning techniques to this data set to analyze COVID-19’s impacts on mental health discourse as a potential proxy for changes in mental health needs.

In the setting of a quarantine, incidence rates rise for mood disorders including acute stress disorder, posttraumatic stress disorder (PTSD), major depressive disorder, and generalized anxiety disorder, as do rates of subclinical mental health deterioration [1]. Early data from the present outbreak gathered in China indicates that rates of depressive symptoms (50.4%), anxious symptoms (44.6%), insomnia (34%), and general distress (71.5%) are especially high among health care workers [2]. Young adults with pre-existing mental and physical health conditions indicate worsening symptoms of depression and anxiety, particularly nonbinary and female young adults [3]. Increased alcohol consumption is linked to isolation, income loss, and adjustments to living with children [4]. The scope of likely mental health deterioration during this pandemic provides an unprecedented need to understand how different mental health cohorts are responding to the outbreak to best design patient assessments and allocate resources. The collective mental health care needs of the general population, which are insufficiently addressed even at baseline, are now increased in the setting of the pandemic. In the context of this limited access to care, it is all the more important to identify subpopulations most impacted by the pandemic to triage resource allocation in an informed manner.

Using natural language processing (NLP) to infer mental states of individuals from their social media posts is a growing field (for reviews, see [5,6]). On Reddit, individuals compose anonymous posts on *subreddit* fora, each with a central topic. Although Reddit posts are not accompanied by formal clinical diagnoses and related covariates, this data set offers several advantages relative to traditional mental health clinical data sets: data is immediately and publicly available, historical data allows for comparisons of multiple time frames, and anonymous and free-form posts create an ecological documentation of vulnerable first-person experiences.

Machine learning models have been created to classify and characterize Reddit posts originating from mental health subreddits [7-10]. Shen and Rudzicz [7] achieved 98% accuracy in separating posts on any of four different anxiety-related subreddits from posts on control subreddits. They used a combination of N-gram language modeling and Linguistic Inquiry and Word Count (LIWC) [11]. LIWC has successfully aided in the detection of depression from Twitter activity with an accuracy of 70% [12] and of bipolar disorder from Reddit posts [8]. These models typically focus on binary classification of posts with respect to a single disorder or subreddit (eg, was the post made on r/Anxiety or a control subreddit).

Visualization and analysis of topics through unsupervised machine learning methods also aid in differentiating between mental health subreddits. Using k-means clustering, analysis of themes present in r/Anxiety, r/Depression, and r/PTSD found that the r/Anxiety and r/PTSD subreddits shared more common terms with each other than with the r/Depression subreddit [13].

Reddit can also help monitor discussions around public health subjects. Using latent dirichlet allocation (LDA), one study identified that Reddit discussions of Ebola focused on health education through posts about best practices and the implications of public health events [14]. LDA uncovers common topics present in text documents, capturing sets of words that typically appear in documents together, which can then be inspected manually to assess common themes across the documents. Combined with an analysis of linguistic changes within mental health subreddits, topic analysis can provide insights into the issues surrounding users during public health crises.

In this paper, we analyze changes and trends in language features during the pandemic to uncover any negative semantic changes, develop machine learning models that successfully classify posts as originating from a particular mental health subreddit to then explain which features characterize each subreddit, understand concerns shared across Reddit independent of the subreddit’s origin using unsupervised machine learning methods, and assess whether subreddits are becoming more similar using supervised dimensionality reduction given the global focus on the pandemic. We expect that quantifying differences between pre- and midpandemic post content and characterizing discussions in the r/COVID19_support group will yield valuable insight into the impact of COVID-19 on mental health and help deploy treatment more effectively. 

## Methods 

### Reddit Mental Health Data set

#### Reddit Users

Demographic information per subreddit is unavailable, but Reddit users collectively are predominantly American (49.9%), male (67%), and young (22%, 18-29 years of age; 14%, 30-49 years of age) [15,16].

#### Data Downloading and Preprocessing

Data was downloaded using the pushshift application programming interface [17]. Posts were extracted from fifteen subreddits focused on specific mental health communities (r/EDAnonymous, r/addiction, r/alcoholism, r/adhd, r/anxiety, r/autism, r/BipolarReddit, r/bpd, r/depression, r/healthanxiety, r/lonely, r/ptsd, r/schizophrenia, r/socialanxiety, and r/SuicideWatch), two broad mental health subreddits (r/mentalhealth and r/COVID19_support), and 11 nonmental health subreddits (r/conspiracy, r/divorce, r/fitness, r/guns, r/jokes, r/legaladvice, r/meditation, r/parenting, r/personalfinance, r/relationships, and r/teaching). Details of data and preprocessing are provided in Multimedia Appendix 1 Methods 1.1.

#### Feature Extraction

The following features were extracted from posts: LIWC (n=62); sentiment analysis (n=4); basic word and syllable counts (n=8); punctuation (n=1); readability metrics (n=9); term frequency–inverse document frequency (TF-IDF) ngrams (256-1024) to capture words and phrases that characterize specific posts; and manually built lexicons about suicidality (n=1), economic stress (n=1), isolation (n=1), substance use (n=1), domestic stress (n=1), and guns (n=1). Trend analysis used all features except TF-IDF; classification and supervised dimensionality reduction used all features, as did unsupervised clustering, and LDA used TF-IDF features. See Multimedia Appendix 1 Methods 1.2 for more details.

### Classification and Feature Importance

#### Training and Testing 

Binary classification was performed on each of the 15 specific mental health subreddits versus a control group (n=2700 each) made of a random sample of the remaining subreddits (first balanced to assure subreddits were equally represented). Only one post per user was used to avoid overfitting. An 80-20 train-test split was used. Two additional test sets were built to test the prepandemic model. One included midpandemic (from March 11, 2020, to April 20, 2020; mean n=504, SD 54.3 posts combined) to measure potential data set shift. A second test set was composed of posts from r/COVID19_support and a control group (n=1574 combined) to measure how they would be classified by our prepandemic model. A weighted F1 was used to measure performance.

#### Models

Our goal was to use the model with the lowest complexity to determine feature importance more directly as long as the model is not considerably outperformed by more complex models. We tested three linear models (stochastic gradient descent linear classifier [SGD] with L1 penalty, SGD with elastic net penalty, and linear support vector machine) along with two more complex tree ensemble classifiers (extra trees and gradient boosting trees).

#### Trend Analysis

We first tracked COVID-19–related tokens (see Multimedia Appendix 1 Table S1) from January 1, 2020, to April 20, 2020, across mental health subreddits. We compared this to the confirmed COVID-19 cases with data obtained from [18]. We then grouped data every 2 days from January 1 to April 20 for every year. For each of the 90 features and 28 subreddits, we fit a linear regression for the average feature value at each time point as a function of time. We applied the Benjamini-Hochberg procedure for multiple hypothesis testing correction with α=.05. We defined change for each feature and subreddit as the slope x R^2^ (ie, the rate of change weighed by the goodness of fit). We tested the amount of absolute change between years by using a Mann-Whitney *U* test.

### Unsupervised Clustering

Prepandemic posts made in 2019 from 15 mental health subreddits were downsampled to a balanced representation (1500 posts/subreddit). The feature set for these posts previously described, including 1024 TF-IDF ngrams, was reduced to 30 principal component analysis (PCA) components. scikit-learn’s SpectralClustering function was used to identify k=20 clusters, using default parameters apart from a nearest neighbors–based (n=10) affinity matrix (selection of k is described in Multimedia Appendix 1 Figure S8). Wilcoxon rank sum tests (with Bonferroni correction) identified cluster-characteristic features for annotating each cluster with a defining theme. Cluster annotation validity was verified through post review. Hypergeometric tests (with Bonferroni correction) identified enrichment of posts from particular clusters on particular subreddits. Posts from the midpandemic data set were processed through the same pipeline. The resulting clusters were compared on the basis of cluster-characteristic features to clusters defined in the prepandemic data set, and the vast majority of clusters were found to have a close match pairing in each time window, as illustrated in Multimedia Appendix 1 Table S4.

### Topic Modeling With Latent Dirichlet Allocation

The *gensim* library was used to perform LDA model estimation, which determined sets of words that appeared frequently together in posts across mental health subreddits. To balance across subreddits, 2700 prepandemic posts from 2019 were sampled from each of the 15 focused mental health subreddits to form our model. Multiple models were created to assess topic stability. A final prepandemic model with 10 topics was chosen to ensure distinct and important topics. A separate model was created with 10 topics using 1300 posts from each midpandemic mental health subreddits, spanning January 1, 2020, to April 20, 2020, to assess any shift in topics. Models were then applied to all subreddits to assess the distribution of the posts across the topics. Models were applied to midpandemic posts between March 16, 2020, and April 20, 2020, to capture topic distributions during the acute phase of the pandemic. Comparison of topic distributions between pre- and midpandemic posts was done with a two-sided Wilcoxon signed rank test with the Benjamini-Hochberg procedure (α=.05) to test whether the incidence of these topics changed across subreddits as a result of the pandemic. Details are provided in Multimedia Appendix 1 Methods 1.3.

### Measuring Similarity Between Subreddits Over Time With Supervised Dimensionality Reduction

With the goal of measuring the similarity between the 15 mental health subreddits as COVID-19 spread, we first reduced posts’ 346D feature vectors to 2D using supervised Uniform Manifold Approximation and Projection (UMAP), a dimensionality reduction technique that can capture nonlinear structures in the data (in comparison to PCA) and that better preserves global structure in comparison to other methods such as t-distributed stochastic neighbor embedding [19]. We then measured the asymmetric Hausdorff distance between subreddits as time progresses to estimate which subreddits are becoming more similar (ie, less distant) to each other. The Hausedorff distance between two clusters is the greatest of all the distances from a point in one cluster to the closest point in the other cluster and, therefore, considers all points in a cluster instead of just a single point such as the centroid as other distances like Euclidean distance would. We balanced subreddits to 1300 posts each spanning from January 1, 2020, to April 20, 2020, grouped in 15-day time windows. This subsampling and dimensionality reduction was repeated for 50 bootstrapping samples, and we took the median distance value. See Multimedia Appendix 1 Methods 1.4 for a test of the precision of this method on 2019 data.

## Results

### Classification and Feature Importance

Models all performed similarly with mean weighted F1 scores between 0.798-0.857 (see Multimedia Appendix 1 Table S1). The SGD L1 (F1=0.851) was chosen for further analysis given that it had the lowest model complexity. Important features are available in Table 1. We then applied this model to midpandemic data (March 11, 2020, to April 20, 2020), and performance changed in several classes (see Multimedia Appendix 1 Table S3). These binary classification models were applied to r/COVID19_support posts to characterize them psychologically (in section r/COVID19_support Characterization).

**Table 1:** Important features for classification (ranked).^a^

Subreddit	Positive coefficients	Negative coefficients
r/EDAnonymous	ed, restrict, purg, bing, calori, LIWC^b^ ingestion, fast, recoveri, eat, ate	bpd, anxieti, addict, diagnos, drug, substance use lexicon, ptsd, LIWC work, LIWC health, med
r/addiction	addict, clean, smoke, rehab, sober, drug, weed, relaps, use, guns lexicon	bpd, diagnos, ptsd, adhd, therapi, isolation lexicon, LIWC hear, therapist, post, LIWC work
r/adhd	adhd, adderal, add, vyvans, focu, forget, final, LIWC work, medic, came	bpd, ptsd, hurt, therapi, guess, suicidality lexicon, fear, bodi, suicid, pain
r/alcoholism	sober, alcohol, drink, withdraw, drunk, LIWC nonfluencies, drank, meet, beer	drug, weight, therapi, adhd, medic, isolation lexicon, notic, attack, dure, addict
r/anxiety	anxieti, wa dead, LIWC negative emotion, LIWC money, anxiou, LIWC motion, LIWC numbers, panic attack, anxious	ptsd, bpd, adhd, LIWC ingestion, LIWC articles article, addict, kill, substance use lexicon, LIWC body, social
r/autism	autism, autist, spectrum, son, game, diagnos, function, diagnosi, explain, interest	ptsd, bpd, addict, LIWC health, disord, adhd, med, 2, stay, guns lexicon
r/BipolarReddit	bipolar, manic, mania, lithium, mood, episod, psychiatrist, hospit, LIWC money, med	adhd, addict, bpd, ptsd, LIWC ingestion, LIWC anxiety, LIWC work, automated readability index, LIWC future tense
r/bpd	bpd, fp, LIWC numbers, LIWC inclusive, LIWC negative emotion, LIWC sadness, bad, drug, LIWC affective processes, feel	ptsd, adhd, weight, LIWC articles article, LIWC health, addict, LIWC 1st pers, anxious, food, isolation lexicon
r/depression	depress, LIWC sadness, LIWC negations, gunning fog index, LIWC positive emotion, LIWC family, cri, LIWC feel, bed, LIWC total pronouns	bpd, symptom, ptsd, adhd, food, isolation lexicon, LIWC conjunctions, diagnos, addict, n sents
r/healthanxiety	cancer, LIWC biological, health anxieti, LIWC health, health, LIWC body, test, fine, LIWC assent, googl	ptsd, adhd, bpd, addict, emot, disord, LIWC 3rd pers, LIWC social processes, social, mental
r/lonely	lone, loneli, isolation lexicon, messag, LIWC certainty, friend, girl, LIWC positive emotion, sit, LIWC religion	LIWC anxiety, ptsd, addict, bpd, suicidality lexicon, symptom, therapist, abus, suicid, med
r/ptsd	ptsd, trauma, flashback, trigger, nightmar, sexual, domestic stress lexicon, abus, tire, guns lexicon	bpd, addict, drink, adhd, LIWC health, isolation lexicon, LIWC work, disord, LIWC certainty, LIWC sadness
r/schizophrenia	schizophrenia, hallucin, delus, schizophren, voic, paranoid, LIWC religion, hospit, ill, LIWC tentative	adhd, ptsd, bpd, addict, abus, flesch kincaid grade level, isolation lexicon, LIWC body, LIWC biological, LIWC health
r/socialanxiety	social anxieti, nervou, walk, awkward, girl, group, convers, speak, face, anxieti	bpd, adhd, ptsd, LIWC health, addict, diagnos, suicid, LIWC sadness, support
r/SuicideWatch	suicidality lexicon, suicid, LIWC negations, death, kill, want die, LIWC sadness, LIWC friends, LIWC money, plan	bpd, symptom, anxious, substance use lexicon, usual, LIWC 2nd pers, smog index, isolation lexicon, weight, attack

^a^Their presence makes it more (positive) or less (negative) likely the classifier will predict the subreddit. Individual word stems are obtained from term frequency–inverse document frequency.

^b^LIWC: Linguistic Inquiry and Word Count.

### Trend Analysis

**Figure 1:**
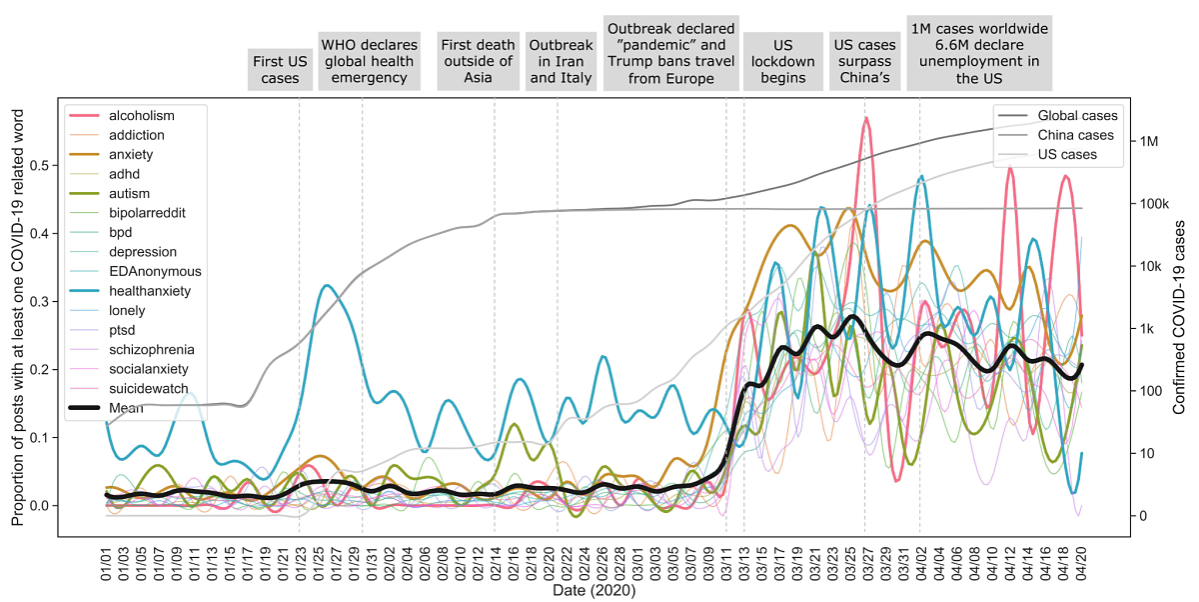
Mention of COVID-19–related words across mental health support groups. Timeline landmarks were chosen from NBC News timeline given that US users are the most prevalent across Reddit. Global, China, and US confirmed COVID-19 cases are displayed. The overall acute rise in COVID-19–related words occurs on March 11, 2020. The correlation between the mean proportion of COVID-19–related posts and global COVID-19 cases is ρ=0.83 (*P*<.001). The health anxiety subreddit has a large increase in COVID-19–related posts almost 2 months before the general increase. r/alcoholism has the most amount of posts related to COVID-19 on March 27. adhd: attention-deficit/hyperactivity disorder; bpd: borderline personality disorder; EDAnonymous: Eating Disorders Anonymous; ptsd: posttraumatic stress disorder; WHO: World Health Organization.

**Figure 2:**
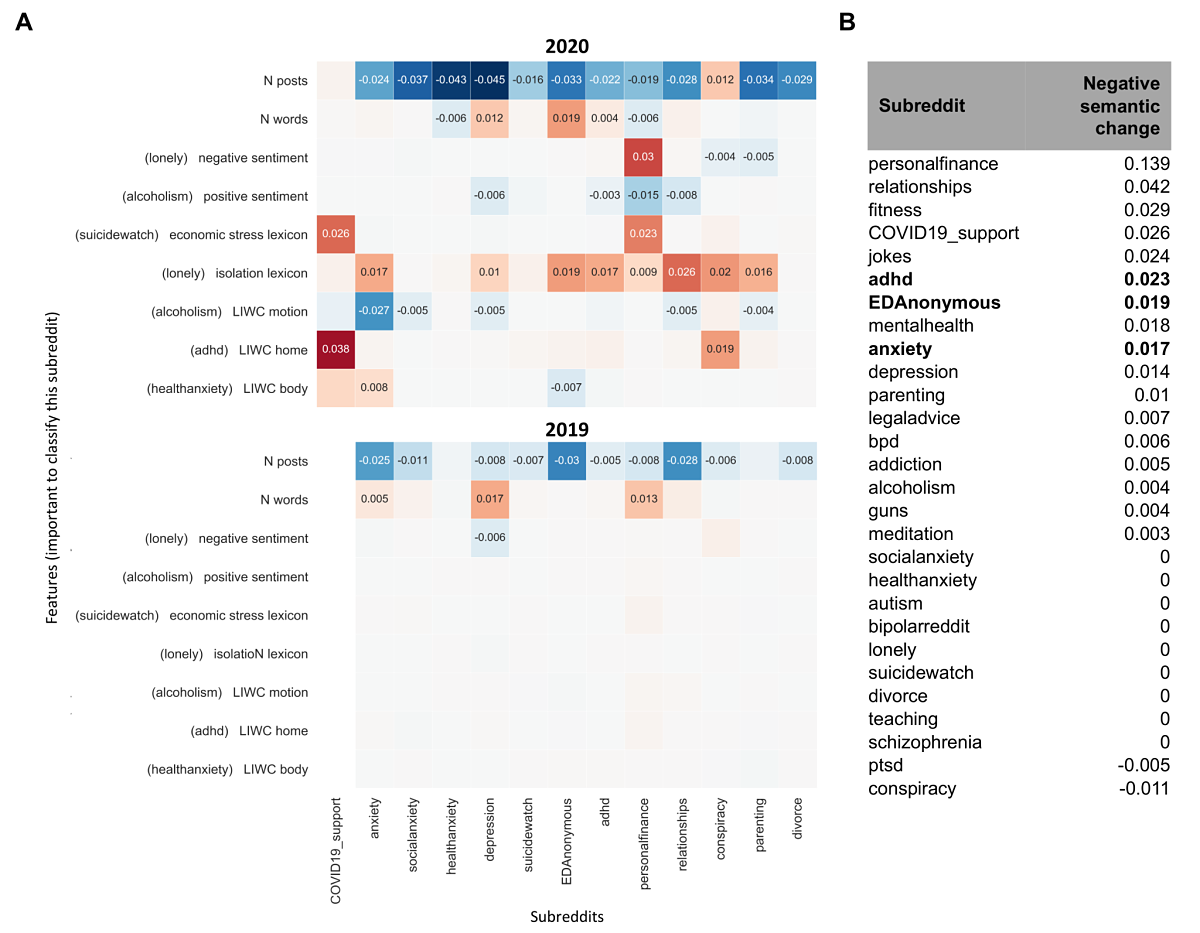
Trend analysis of linguistic features over time. A) Significant change in average feature values from January to April 2020 and 2019 across subreddits. COVID19 support subreddit was created in 2020 and, therefore, does not appear in 2019. Features important for classifying a subreddit are added to the y-axis. Change is defined by slope x R^2 (ie, increases have a positive slope and tend toward red, decreases have a negative slope and tend toward blue). Significant trends after multiple comparison correction on full results are displayed. There is significantly more absolute change in 2020 than in 2019 (*P*<.001) and 2018 (*P*<.001). B) Rank of subreddits by the amount of negative semantic change throughout COVID-19 (January 1, 2020, to April 20, 2020) across significant full results. In bold are the mental health subreddits with the most negative semantic change using the following features with emotional valence: negative sentiment; the lexicons about economic stress, isolation, substance use, guns, domestic stress, and suicidality; LIWC measures of anger, anxiety, death, negations, negative emotion, and sadness; and three positive features inversely weighed, compound sentiment, positive sentiment, and positive emotion. r/ptsd and r/conspiracy decreased in negative semantic features. adhd: attention-deficit/hyperactivity disorder; bpd: borderline personality disorder; EDanonymous: Eating Disorders Anonymous; LIWC: Linguistic Inquiry and Word Count; ptsd: posttraumatic stress disorder.

See Figure 1 for the proportion of posts about COVID-19 in each support group. For a measure of how much of each post was about COVID-19, see Multimedia Appendix 1 Figure S3. For an example of how trends were computed for a single feature and subreddit, see Multimedia Appendix 1 Figure S4. Figure 2 shows a subset of the trends and most negative semantic change (ie, sum of change in negative semantic features). Throughout many subreddits, we found significant increases in the use of tokens related to isolation (eg, “lonely,” “can’t see anyone,” “quarantine”), economic stress (eg, “rent,” “debt,” “pay the bills”), and home (“fridge,” “pet,” “lease”), and a decrease in the lexicon related to motion (eg, “walk,” “visit,” “travel”), all consistent with the type of changes many are facing during the ongoing pandemic. See Multimedia Appendix 1 Figures S5-S7 for full results (90 features in 28 subreddits). See Textbox 1 for examples of posts that score high on important features and display risky behavior.

**Example of posts that score high on a subset of important features.**

**COVID-19 lexicon**

**r/healthanxiety (January 19, 2020):** “I’ve been seeing a lot of news stories today about an incurable, lethal disease originating in China spreading to other countries. I know the media often over exaggerates things like this, but WHO is debating whether or not to declare this an international emergency. Nothing triggers my anxiety worse than things like this, if anyone has any advice on how to cope/can inform me on it I would really appreciate it!”

**Linguistic Inquiry and Word Count (LIWC) achievement**

**r/addiction (March 20, 2020):** “Anyone else relapse during quarantine? Looking for others who are going through this and would like to swap experience, strength and hope?...all my meetings have been canceled”

**LIWC affective processes**

**r/bpd (March 27, 2020):** “...I posted this in another group but I’m really struggling right now. I really wish I could get into a DBT group but now I’m so confused with all of this virus stuff. Does anyone have effective tips for dealing with splitting? Sometimes I will be upset with my S/O for hours or days and I don’t even know why at a certain point. I get so manipulative and I really hate it. Does anyone else get like this?”

**LIWC ingestion**

**r/EDAnonymous (March 19, 2020):** “Quarantine is slowly chipping away at my recovery. I can't help but feel like this is the perfect time to fast or restrict since no one's monitoring my meals. Nobody at school noticing. I'm skipping lunch for the 4th day in a row or only eating celery. I was doing so well!!!...”

**LIWC money and guns lexicon**

**r/SuicideWatch (March 29, 2020):** “Suicide is too expensive with increasing gun prices I've been watching gun deals to see if I could afford something that was quick and surefire, but covid has made guns more expensive with greater wait times.”

**LIWC negations**

**r/depression (March 21, 2020):** “I can’t do it If things don’t get back to normal in a few weeks I want to kill myself. The only thing I was excited about was starting a new job and I can’t anymore because of all the coronavirus shut downs. I’m going crazy I don’t have anyone to talk to and I just can’t do this anymore”

**LIWC see**

**r/socialanxiety (April 13, 2020):** “Now that everyone is wearing face masks, I suddenly don't have trouble making eye contact with people anymore. I might keep wearing a mask out in public after the quarantine is over. I really like the confidence it gives me. I don't have to worry about what my facial expression is or looks like, I don't have to worry about smiling, everyone looks the same around me.”

**Economic stress lexicon**

**r/COVID19_support (March 10, 2020):** “Would I be allowed a temporary paid Leave of Absence by my psychiatrist from my unionized supermarket due to COVID19? It’s causing me major anxiety, everyone else is calm but it sadly compromises my calmness. I am diagnosed with schizoaffective disorder/bipolar. There’s only one confirmed case in my county, but still. School for me, may be canceled. How do I handle working my part time job?”

**Negative sentiment**

**r/adhd (March 20, 2020):** “Drug interactions with COVID-19?? I just read about methylphenidate being directly related to a lower white blood cell count/ worse immune system...AND it can cause high blood pressure. I’m hyper-focused on this virus and i’m terrified that Concerta is putting me more at risk. Anyone else in the same boat?”

**LIWC health**

**r/conspiracy (March 13, 2020):** “...How has the coronavirus been treated? Iodine and vitamin C. How has radiation sickness been treated? Iodine and vitamin C. China and Italy lead the world in radioactive 5G cell towers. 5G operates on the same frequency and wavelength as military grade weapon technology. Connect the dots.”

### Unsupervised Analysis

#### Unsupervised Clustering Uncovers Post Language Themes

**Figure 3:**
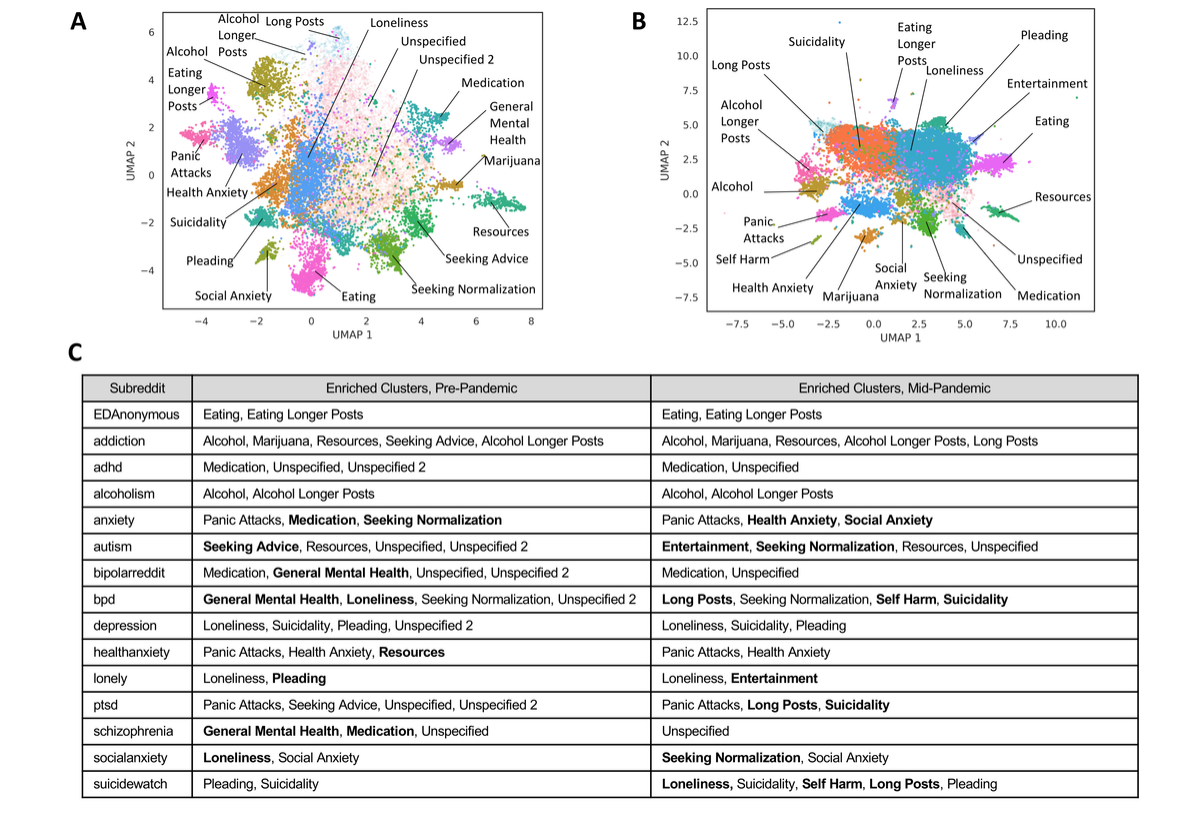
Unsupervised clustering reveals post groupings with representation across mental health subreddits. A) Unsupervised clustering of pre-pandemic (year 2019) posts from 15 mental health subreddits, presented in 2D UMAP space. Posts in two thematically-related, adjacent clusters were collapsed into a single “Resources” cluster. Three clusters could not be assigned identifiable themes. Two of these—annotated as ”Unspecified”—were the largest clusters in the dataset, containing 6329 and 4272 total posts, respectively, while the next largest cluster contained 1620 posts. The other cluster without an identifiable theme was characterized by very long posts. This ”Long Posts” cluster had an average post length of 886 words, while the cluster with the next most lengthy posts had an average post length of 554 words. As a result, this “Long Posts” cluster had an overwhelming number of cluster-characteristic text features, which made any core linguistic theme poorly discernible. The identified clusters were not an approximation of post subreddit of origin, as demonstrated by several metrics quantifying the lack of correspondence between cluster labels and post subreddit of origin: Homogeneity (0.20), Completeness (0.22), V-measure (0.21), and Adjusted Rand-Index (0.08). B) Unsupervised clustering of mid-pandemic posts using the same process resulted primarily in replication of cluster annotations observed in the pre-pandemic data, with a few clusters (e.g., Seeking Advice) detected only in the pre-pandemic clustering and a few (e.g., Entertainment) detected only in the mid-pandemic clustering. Two clusters increased notably in size in the mid-pandemic clustering: Suicidality (204% increase in number of posts) and Loneliness (233% increase in number of posts). C) Enrichment of clusters on mental health subreddits during the pre-pandemic period and the mid-pandemic period, using clusters detected during each time period, respectively. Associations were assessed with hypergeometric tests, and those displayed here passed strict Bonferroni correction for multiple hypothesis testing. Associations present only for the pre-pandemic or only for the mid-pandemic time period are shown in bold.

Clustering of prepandemic posts (Multimedia Appendix 1 Figure S8, Figure 3) highlights that language use across subreddits represents a continuum but contains meaningful axes of variation reflected by the proximity of clusters for closely related conversation topics. Cluster-distinguishing terms (Multimedia Appendix 1 Table S4) used to assign annotations reveal that some clusters are characterized by discussion of particular mental health concerns (eg, “Suicidality”) while others are characterized by post tone (eg, “Seeking Normalization”).

We identified 47 cluster-subreddit pairings (Figure 3C) for which posts from the given cluster were enriched on the given subreddit. Expected pairings were recapitulated and additional associations of interest were revealed. For example, r/addiction is enriched for posts from the “Substance Use Alcohol” and “Substance Use Marijuana” clusters, as expected. It is also one of few subreddits enriched for posts from the “Resources” and “Seeking Advice” clusters. Although cluster-subreddit associations are present, every cluster contains posts from ≥12 subreddits, emphasizing the value of clustering to define categories that span subreddits. Clusters defined by tone and unspecified clusters contain the greatest diversity of post representation across subreddits, quantified by the Shannon Index (Multimedia Appendix 1 Table S5). The distribution of posts across subreddits within four example clusters in Multimedia Appendix 1 Figure S9 illustrates the subreddits with greatest representation in the medication cluster (r/adhd, r/BipolarReddit, r/schizophrenia, and r/anxiety), the pleading cluster (r/SuicideWatch, r/lonely, r/depression, and r/addiction), the social anxiety cluster (r/socialanxiety, r/anxiety, and r/autism), and the suicidality cluster (r/SuicideWatch, r/depression, and r/bpd).

#### LDA Topic Modeling on Multiple Time Frames

**Figure 4:**
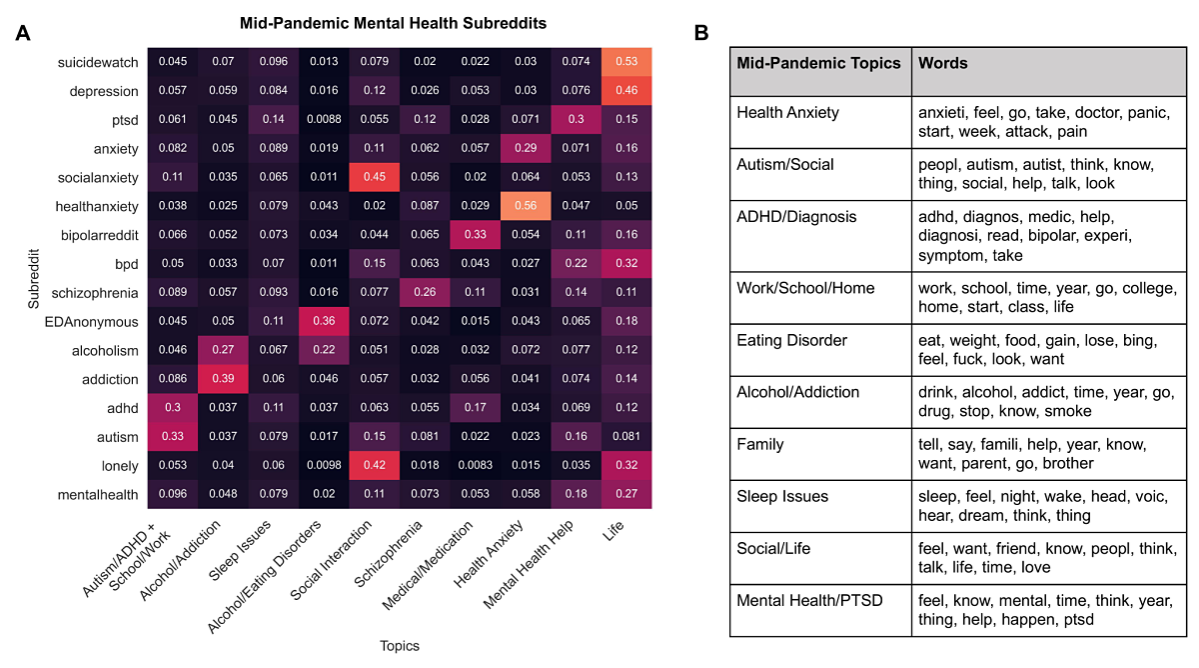
Latent dirichlet allocation (LDA) reveals prominent topics in mental health subreddits. A) Distribution of midpandemic posts from 15 mental health subreddits across 10 topics extracted using LDA on prepandemic mental health subreddit posts. Topic distribution was assessed for midpandemic posts between March 16, 2020, and April 20, 2020, to capture the phase of the pandemic right after stay-at-home orders had been announced or enacted for many areas in the United States. Inspection of the topic distribution indicated that there was minimal shift in most topics for all subreddits between the pre- and midpandemic time frames. We tested changes in topic distributions across all 27 subreddits using a Wilcoxon signed rank test (COVID19_support was not available during 2019). B) Manually labelled topics and the top 10 terms associated with each topic derived from an LDA model created on midpandemic subreddit posts. ADHD: attention-deficit/hyperactivity disorder; PTSD: posttraumatic stress disorder.

Figure 4 shows the distribution of posts in midpandemic mental health subreddits over the 10 topics extracted using LDA on prepandemic mental health subreddits as well as the 10 topics extracted using LDA on the midpandemic mental health subreddits. The topic number was chosen to include distinct yet important topics. Topics extracted from the prepandemic LDA model (x-axis of Figure 4A, and Multimedia Appendix 1 Table S6) largely matched the expected topics from the prepandemic subreddits. Topics emerged related to “Alcoholism and Addiction,” “Health Anxiety,” “Alcoholism and Eating Disorder,” “Schizophrenia,” and “ADHD and Autism,” corresponding to specific subreddits. The attention-deficit/hyperactivity disorder (ADHD) and autism token words were also combined into the same topic with tokens related to school and work, and did not separate even in models with a larger number of topics. More general topics such as “Social Interaction,” “Life,” and “Mental Health Help” also emerged, which captured common topics in subreddits such as r/SuicideWatch, r/depression, r/lonely, and r/mentalhealth. Compared to the prepandemic topic model, the midpandemic LDA model splits the autism and ADHD tokens, includes a topic on family, and includes a topic with a PTSD token. Multimedia Appendix 1 contains the distribution of prepandemic model topics across mental health subreddits (Multimedia Appendix 1 S10) and across non–mental health subreddits (Multimedia Appendix 1 Figure S11) in both pre- and midpandemic time frames. Wilcoxon signed rank tests of distributions of the pre- and midpandemic posts over prepandemic model topics implies there was an increase in the “Health Anxiety” (*P=.*008) topic and in the “Life” topic (*P=.*01), as well as a decrease in the “Alcoholism and Addiction” topic (*P=.*004), while the remainder of the topics showed no significant change in distribution after multiple comparison correction.

### r/COVID19_support Characterization

**Figure 5:**
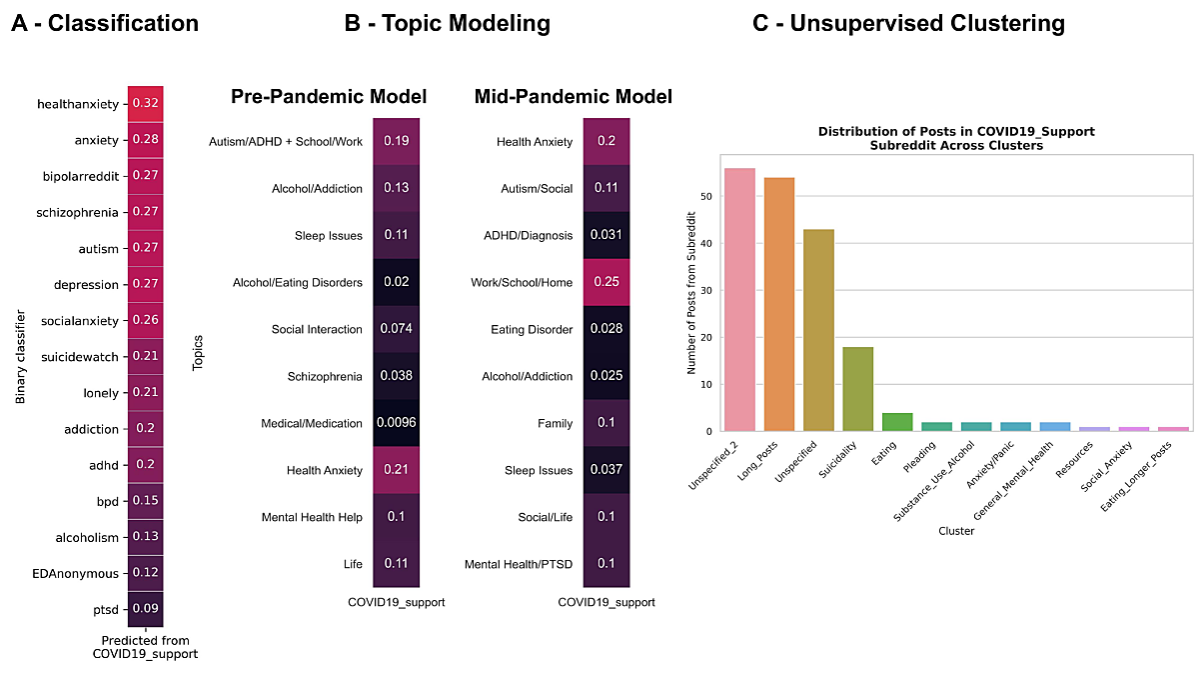
Characterization of r/COVID19_support through supervised and unsupervised methods. A) Proportion of r/COVID19_support posts (March 11 to April 20, 2020) that each binary classifier trained on prepandemic data detects. B) Distribution of prepandemic model topics (left) and midpandemic model topics (right) for posts in r/COVID19_support, highlighting prominent topics in the posts, such as health anxiety and issues in school, work, and home scenarios. The distribution of topics indicate common themes of pain points, which could help guide the medium and content of mental health resources. C) Distribution of unsupervised cluster representation among posts from r/COVID19_support. Although many posts were assigned to unspecified clusters, the substantial portion of posts assigned to the suicidality cluster is notable. ADHD: attention-deficit/hyperactivity disorder; EDanonymous: Eating Disorders Anonymous; PTSD: posttraumatic stress disorder.

Figure 5 characterizes the new r/COVID19_support subreddit through supervised and unsupervised methods. Health anxiety emerged as a major concern through classification (ie, r/healthanxiety was classified most prevalently) and topic modeling, while unsupervised clustering revealed a substantial portion of posts were assigned to the suicidality cluster.

### Measuring Similarity Between Subreddits Over Time With Supervised Dimensionality Reduction

**Figure 6:**
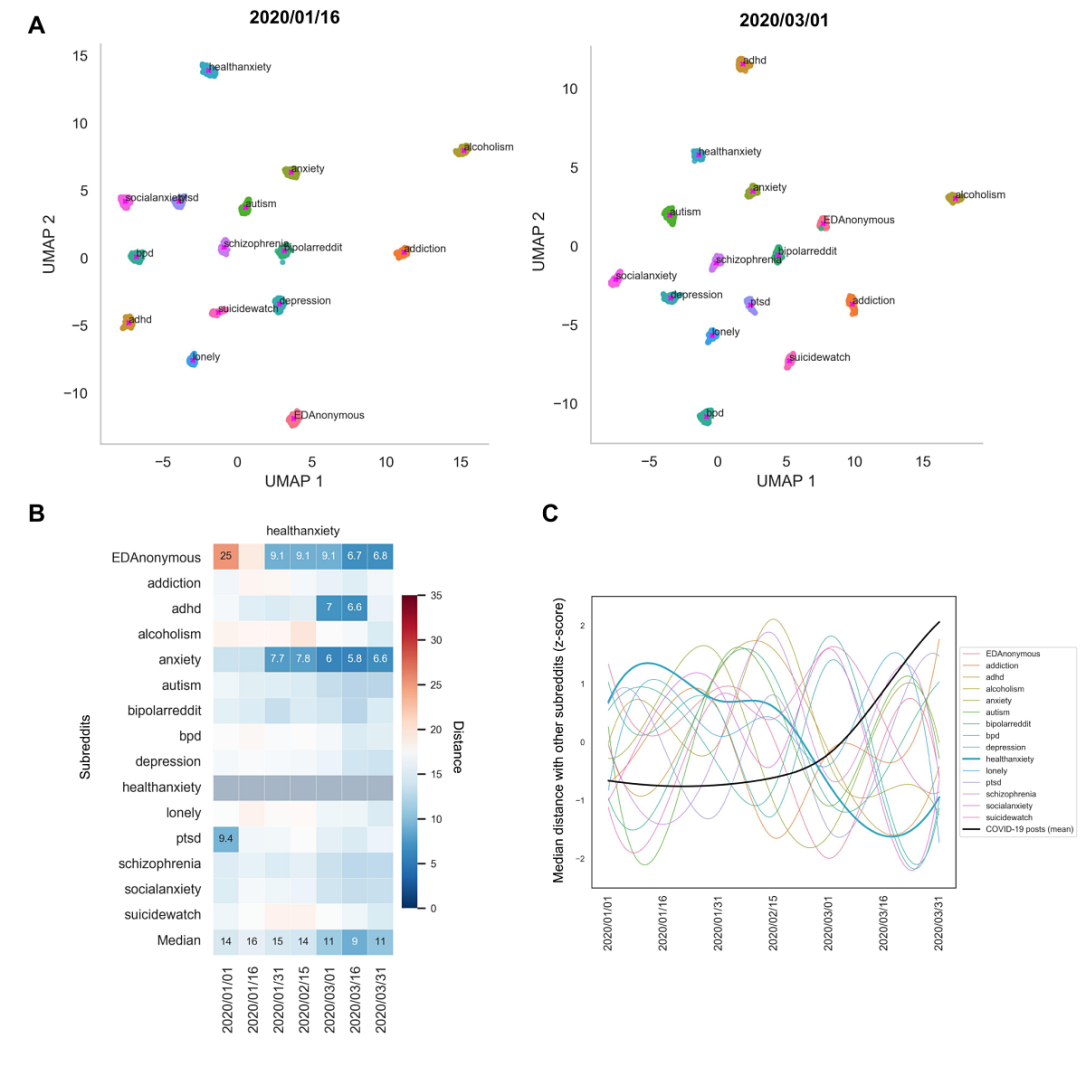
Supervised dimensionality reduction to measure how certain subreddits are becoming more or less similar over time. A) Supervised dimensionality reduction of posts within 15-day time windows with starting day displayed (r/healthanxiety becomes more similar to other subreddits). B) Median pairwise distance with r/healthanxiety for each time window over 50 bootstrapping samples displaying only extreme values with regard to normal 2019 fluctuations (top and bottom 5th percentiles), which indicates they are less likely to be part of normal fluctuations in distance. C) The median distance across all subreddits (last row in B) shows subreddits becoming more similar to r/healthanxiety during the increase in COVID-19–related posts (Figure 2 mean values were split into 7 time windows to match subreddit trends, and the mean was taken for each window). r/healthanxiety is the only trend that significantly correlates with COVID-19 posts after Benjamini-Hochberg multiple comparison correction (ρ=–0.96, *P*<.001). adhd: attention-deficit/hyperactivity disorder; bpd: borderline personality disorder; EDanonymous: Eating Disorders Anonymous; ptsd: posttraumatic stress disorder; UMAP: Uniform Manifold Approximation and Projection.

The highest silhouette score (0.93) was obtained with n neighbors=200, min dist=0.0, and cosine distance. Using UMAP and true labels, we reduced feature sets for each post to 2D and measured the directed Hausdorff distances between the clusters to quantify which subreddits are converging or diverging as the pandemic advances (see Figure 6 and Multimedia Appendix 1 Figure S12 for full results).

## Discussion

We applied an array of NLP techniques including statistical analysis of feature trends, supervised learning, interpretability, and unsupervised learning to measure how COVID-19 may have impacted different mental health support groups. Overall, we discuss each analysis in turn, focusing on clinical takeaways.

### Trend Analysis

In our results, r/healthanxiety had several spikes in pandemic-related posts in January before other subreddits were posting about a possible pandemic (see example of post in Textbox 1). This evidence supports concerns regarding the prolonged stress that people with health anxiety may be experiencing. We then evaluated the nature of linguistic changes in mental health subreddits as the pandemic spread. Strikingly, many linguistic features significantly changed from January to April of 2020 than in the same months of previous years (see Multimedia Appendix 1 Figure S5 for full results). The amount of posts significantly decreased in multiple subreddits in 2020, as tends to happen during that time period, but the decrease was often more than in previous years. One potential reason is that some users may avoid social media since they perceive increased anxiety from news and discussions, and want to protect their mental health. However, this could be a concern if individuals are not using these subreddits to seek support and have limited access or barriers to other support structures including psychotherapy. Interestingly, the amount of posts significantly grew for r/conspiracy, which is consistent with findings from other analyses [20]. A sign of concern comes from the increase in negative semantic features for certain subreddits, the highest of which belonged to the groups for ADHD, eating disorders, anxiety, and depression within the mental health subreddits and personal finance, relationships, and fitness within the non–mental health subreddits. Regarding ADHD, some parents in France of children and adolescents diagnosed with ADHD reported increased hyperactivity and inattention, while other parents reported symptomatic improvement [21]. There is also evidence that individuals with eating disorders are experiencing worse symptoms and heightened risk for relapse, hypothesized to stem from limited care access, less structure in daily activities, and decreased social support [22], and captured in the example in Textbox 1. We found an increase in the use of body-related words for r/anxiety and a decrease in r/EDAnonymous and r/fitness. Pandemic-related features (motion, isolation, economic stress, home) increased or decreased across some subreddits in the direction that would be expected for a pandemic, and the amount of change provides further evidence of concerns or lack thereof. For instance, although the isolation feature significantly increased for most subreddits, it increased most for r/anxiety and did not increase for r/socialanxiety (ie, users write less about isolation and loneliness; see r/socialanxiety example in Textbox 1).

### Classification and Feature Importance

We successfully developed binary models to classify 2019 posts originating from a given mental health subreddit as distinct from posts originating from other mental health–related subreddits. We then used these models to classify r/COVID19 support posts and identify the general distribution of complaints as well as identify posts potentially containing at-risk behavior (eg, from r/SuicideWatch). By leveraging interpretable linear methods, we were able to establish features key to distinguishing mental health subreddits from one another (see Multimedia Appendix 1 Tables S4 and S5, and the y-axis of Figure S5), which helps understand how different mental health concerns may manifest in language. Some of the most interesting top important features used to classify each subreddit were gunning fog index (ie, how readable the text is) for r/depression, the tokens “cancer” and “google” for r/healthanxiety, domestic stress and guns lexicons for r/ptsd, LIWC “religion” and number of long words for schizophrenia; LIWC see (eg, “picture,” “screen,” “stare”) for r/socialanxiety, and LIWC money and certainty for r/SuicideWatch (see Textbox 1 for more examples).

### Unsupervised Methods: Topic Modeling and Clustering

We established important conversation topics that span across mental health support subreddits through unsupervised methods (LDA and clustering). Unsupervised clusters such as seeking advice, resources, seeking normalization, suicidality, and medication varied in representation across the mental health subreddits and provided insight into the forms of discussion occurring. This cluster structure has utility for assessing changes in discussion on mental health and for analyzing subreddits like r/COVID19_support, on which many posts were found to map to the suicidality cluster. r/bpd and r/ptsd became significantly enriched for posts from the suicidality cluster in the midpandemic data set. The r/anxiety subreddit became significantly enriched for posts from the health anxiety cluster, which captures the general theme of heightened health anxiety. Topic modeling found an increase in the distribution of the health anxiety topic across midpandemic posts and highlighted changes in topics between pre- and midpandemic time frames, such as the introduction of the topics social interaction and mental health help. Additionally, a large number of posts in the r/COVID19_support group were identified to relate to the health anxiety topic and to voice concerns with daily living at home, school, and work. Posts in these topics and clusters could be important for subreddit moderators to track as they seek to cultivate a culture of support and provide effective assistance to authors in crisis (eg, tracking clusters like seeking advice, medication, suicidality, and resources) and understand the chief concerns of their communities (eg, tracking topics like sleep issues and social interaction).

### Measuring Similarity Between Subreddits Over Time With Supervised Dimensionality Reduction

Psychiatric care for patients during the pandemic should be informed by an understanding of possible convergence among some disorders, which could merit a blending of standard treatment approaches, and of possible separation of certain clusters from the rest, which could identify an at-risk population. Overall, the more users were posting about COVID-19, the more similar subreddits became to r/healthanxiety (see Figure 5C). The r/healthanxiety similarity to other subreddits was the only subreddit that significantly correlated with the rise in COVID-19–related posts. Interestingly, the subreddits that became most negative per trend analyses, r/ADHD, r/EDAnonymous, and r/Anxiety, also became most like r/healthanxiety during the general spike of COVID-19–related posts during March 2020. These results suggest a clinically testable hypothesis that the symptom of health anxiety may have increased most in the psychiatric populations corresponding to these three subreddits.

### Convergent Findings Across Analyses: Health Anxiety and Suicidality

Our findings suggest the pandemic may have induced health anxiety among several mental health and non–mental health communities given that posts on r/COVID19_support were classified most frequently as belonging to r/healthanxiety, midpandemic posts from the r/anxiety subreddit became significantly enriched for posts from the health anxiety cluster, LDA topic analysis found the health anxiety topic significantly increased in the midpandemic posts compared to the prepandemic posts, and supervised dimensionality reduction revealed that the more users posted about COVID-19 the more similar subreddits became to r/healthanxiety.

Suicidality was another concerning theme given that unsupervised clustering revealed the suicidality cluster doubled in size and a new cluster surrounding self-harm emerged. Notably, a substantial portion of posts from r/COVID19_support were assigned to the suicidality cluster, and two subreddits (r/bpd and r/ptsd) became significantly associated with the suicidality cluster during the pandemic. Furthermore, 26% of posts from COVID19_support were classified by r/SuicideWatch by its binary classifier.

### Limitations

Our study population is not characterized with formally documented clinical diagnoses, although some post authors make diagnostic claims. For example, 5% of posts in our full 2019 data set are authored by individuals who make a diagnostic claim (eg, “I have obsessive compulsive disorder”). If further application of NLP techniques can expand the set of post authors with high likelihood of clinical diagnoses, analyses could be restricted to that subset of authors without loss of sufficient post volume. Furthermore, although linguistic changes occurred during the pandemic, in this study we do not causally link any individual changes to specific events. Finally, in our study, it is possible that post authors who are more stable in their mental health state and would, therefore, have contributed more consistent post content between the prepandemic and midpandemic periods, decrease their participation in these support fora as a means of coping with the stressors of the pandemic. Alternatively, it is also possible that post authors who are more dynamic in their mental health state, and would therefore have contributed to more dramatic differences in post content between the prepandemic and midpandemic periods, decrease their participation in these support fora as a means of coping. Both forms of selection bias are possible and represent a limitation of our observational study design.

### Future Directions

Extremely risky behavior is common content in these posts, including asking for advice for restricting food in r/EDAnonymous or planning suicide in r/SuicideWatch; therefore, more clinical attention is urgently needed to provide effective resources to Reddit users. Furthermore, understanding the nature of posts from subreddits other than r/SuicideWatch (including r/COVID19_support) that were classified as r/SuicideWatch posts or that belong to the suicidality cluster demands further research. Critically, these posts were made on subreddits without the policies and response systems used by moderators on r/SuicideWatch itself and whose authors may benefit from urgent intervention. NLP applied to Reddit posts could help direct users to tailored resources or more ideal subreddits where they are more likely to receive support and help with the triage of moderator responses. For example, authors of posts in the seeking normalization cluster may be looking for solidarity whereas authors of posts with high use of the sleep issues LDA topic may benefit from sleep-related advice. These analyses of Reddit could also be followed by related analyses of posts on Twitter, which enables some geolocation but restricts anonymity and post length. Geographic information could, for example, be used to characterize the impact on mental health of specific policy developments like the imposition of statewide lockdowns that varied in time across geographic areas. Taking trend analysis and supervised dimensionality reduction, one can track subreddits that did not change. More research is needed to understand if certain groups were more resilient to the pandemic. Ultimately, we have found many linguistic patterns for specific mental health groups, and these patterns could be studied further in clinical settings that include formal diagnoses and more extensive covariate information (eg, racial or socioeconomic background).

### Conclusions

We performed successful classification among mental health subreddits and identified important features to understand how each mental health problem may manifest in language. We tracked features across time and observed the largest negative semantic changes during the COVID-19 pandemic for r/ADHD, r/EDAnonymous (eating disorders), and r/Anxiety, which were the same groups that become most similar to r/healthanxiety during the rise of COVID-19 posts in March. Understanding linguistic features that distinguish these communities and how language use has changed during the pandemic has generated several important hypotheses for evaluation in clinical settings that may help inform the provision of responsive care. Importantly, different analyses found increases in health anxiety and suicidality. We hope this work will bring attention to these large online communities’ needs and concerns since Reddit may be the first path to treatment for many online users, and these subreddits currently lack sufficient resources. We have helped to define some of these concerns through multiple methods such as feature importance (eg, the guns lexicon is important for classifying r/ptsd), trend analysis (eg, decreased use over time of the motion lexicon in r/anxiety), unsupervised clustering (eg, a substantial number of posts on COVID19_support mapped to a Suicidality post cluster and subreddits show discussion of topics distinct from the overall subreddit theme), topic modeling (eg, the high rate of concerns regarding home, work, and school on r/COVID19_support). We further hope that insights from this work will deepen our understanding of mental health challenges during the pandemic, inform the provisioning of appropriate therapeutic resources, and inspire greater use of NLP for the inspection of world-changing events such as epidemics, elections, and protests.

## Appendix

Multimedia Appendix 1 Supplementary materials.

## Abbreviations

ADHD: attention-deficit/hyperactivity disorder
LDA: latent dirichlet allocation
LIWC: Linguistic Inquiry and Word Count
MIT: Massachusetts Institute of Technology
NLP: natural language processing
PCA: principal component analysis
PTSD: posttraumatic stress disorder
SGD: stochastic gradient descent linear classifier
TF-IDF: term frequency–inverse document frequency
UMAP: Uniform Manifold Approximation and Projection
