# Supplementary material for: Natural Language Processing Reveals Vulnerable Mental Health Support Groups and Heightened Health Anxiety on Reddit During COVID-19: Observational Study
Source: J Med Internet Res. 2020 Oct 12;22(10):e22635. doi: 10.2196/22635 (PMC7575341; doi:10.2196/22635)
Supplement: Multimedia Appendix 1 [file jmir_v22i10e22635_app1.pdf]

Low, D. M., Rumker, L., Talker, T., Torous, J., Cecchi, G., & Ghosh, S. S. (2020). Natural Language Processing Reveals Vulnerable Mental Health Support Groups and Heightened Health Anxiety on Reddit during COVID-19: Observational Study. *Journal of Medical Internet Research.*

1. Methods

1.1. Data downloading and preprocessing

Posts were extracted from the 28 subreddits listed in the main body, including 15 mental health subreddits, 11 non-mental health subreddits, and the broad mental health subreddits, r/mentalhealth and r/COVID19_support. Only original posts and not comments were included. Only posts including a title and a body were included to exclude posts with just images. Titles and bodies of posts were concatenated. Posts were filtered to only be in English. We only kept the first post from each user in each timeframe and to remove posts from bots and ads. Posts from the following timeframes were downloaded:

- **Midpandemic data (January to April 2020):** all posts on each subreddit were extracted between the dates of 2020-01-01 and 2020-04-20. Unique users: 320,364.
- **Prepandemic data:** all posts made between 2018-11-01 and 2019-11-01 were extracted. Unique users: 327,289.

Posts were either randomly downsampled to 30,000 per subreddit, or all included, with a maximum of 1000 posts extracted per day following pushshift.io limitations. Classification, unsupervised clustering and topic modeling used prepandemic data and midpandemic data. Supplement Figure 1 in provides histograms of how many posts were extracted from each subreddit for the time-frames used in analysis.

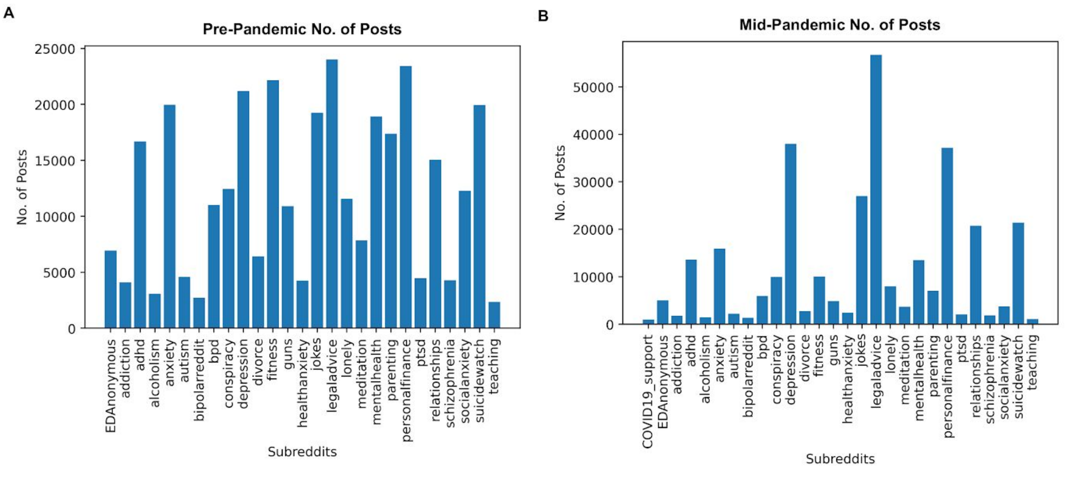

Figure S1: Distribution of extracted posts over all subreddits. The number of posts extracted from each subreddit in our data set for A) prepandemic and B) midpandemic timeframes.

The trend analysis used midpandemic data and the 2019 and 2018 additional control datasets:

- **2019 control for midpandemic (January to April 2020):** all posts made between 2019-01-01 and 2019-04-20 were extracted. Unique users: 282,560.
- **2018 control for midpandemic (January to April 2020):** all posts made between 2018-01-01 and 2018-04-20 were extracted. Unique users: 177,089.

Unique users across all time windows (pre and 2019 overlap): 826,961.

1.2. Feature extraction

The following features were extracted from each post (resulting dimensions): VADER sentiment analysis (3) [1]; Textacy basic word and syllable counts (8) [2]; Textacy readability metrics (9); Linguistic Inquiry and Word Count, which includes words scored by their belonging to different semantic and grammatical categories such as ’body’ or ’pronouns’ (62) [3]; TF-IDF unigrams and bigrams (256-1024 depending on analysis; see below); punctuation count (1); and the following manually built lexicons: suicidality (1); economic stress (1); isolation (1); substance use (1); domestic stress (1); guns (1) (see Table S1 for tokens that make up the lexicons). Lexicon counts were normalized by the amount of words in each post. For LIWC extraction and TF-IDF calculation, all words were lemmatized and stemmed using the NTLK package. Furthermore for TF-IDF calculation, English stopwords and words with three or fewer characters were removed from posts. Further preprocessing depended on the analysis:

Classification: all features were used but TF-IDF (256 features) was fit on the train set and transformed on the test set separately to avoid overfitting.

Trend Analysis: all features except TF-IDF were used for Figures 2, S5, S6, S7. An additional feature quantifying the presence of tokens from a COVID-19 lexicon (see Table S1) was computed for Figures 1 and S3.

LDA: After utilizing the same preprocessing steps as TF-IDF, a dictionary was created with the top 100,000 unigrams and bigrams from all of the posts such that each dictionary token appeared in more than 0.1% of the training posts but less than 50% of the posts.

Unsupervised clustering: Features used for unsupervised clustering included the top 1024 TF-IDF ngrams as well as the non-TF-IDF features listed above.

Supervised dimensionality reduction: we used all features including 256 features for TF-IDF.

Theme	Terms
Economic Stress	Unemploy, economy, rent, mortgage, evict, enough money, more money, pay the bills, owe, debt, make ends meet, afford, save enough, salary, wage, income, job, eviction
Isolation	alone, lonely, no one cares about me, no one cares, can't see anyone, can't see my, i miss my, i want to see my, trapped, i'm in a cage, lonely, feel ignored, ignoring me, ugly, rejected, avoid, avoiding me, am single, been single, quarantine, lockdown, isolation, self-isolation
Substance Use	smoke, cigarette, tobacco, wine, drink, beer, alcohol, drug, opioid, cocaine, snort, vodka, whiskey, whisky, tequila, meth
Guns	gun, pistol, revolver, semiautomatic, rifle, shoot, firearm, semi-automatic
Domestic Stress	divorce, domestic violence, abuse, yelling, fighting with me, we're fighting, single mom, single dad, single parent, hit me, slapped me, fighting, fight
Suicidality	commit suicide, jump off a bridge, i want to overdose, i'm a burden, i'm such a burden, i will overdose, thinking about overdose, kill myself, killing myself, hang myself, hanging myself, cut myself, cutting myself, hurt myself, hurting myself, want to die, wanna die, don't want to wake up, don't wake up, never want to wake up, don't want to be alive, want to be alive, wish it would all end, done with living, want it to end, it all ends tonight, live anymore, living anymore, life anymore, be dead, take it anymore, end my life, think about death, hopeless, hurt myself, no one will miss me, don't want to wake up, if i live or die, i hate my life, shoot me, kill me, suicide, no point
COVID-19	corona, virus, viral, covid, sars, influenza, pandemic, epidemic, quarantine, lockdown, distancing, national emergency, flatten, infect, ventilator, mask, symptomatic, epidemiolog, immun, incubation, transmission, vaccine

Table S1: Manually constructed lexicons. Developed to assess the prevalence of tokens related to these topics in all of the subreddits.

1.3. Latent Dirichlet Allocation Topic Modeling on Pre and MidPandemic Subreddits

The dictionary created as described in Section 1.2 was applied to all posts to create a bag- of-words corpus which was used to create an LDA model using 25 passes and 3 workers. Models with 5, 10, 15, 20, and 25 topics were created. Models were also generated multiple times with different subsamples of posts to assess stability of topics. A manually chosen LDA model with 10 topics was then applied to all posts across all subreddits (mental health and non-mental health) to assess the distribution of topics, allowing for comparison between the distribution of posts prepandemic vs midpandemic. A manually chosen LDA model created on midpandemic data was applied to posts from r/COVID19_support to assess any change in topic distribution.

1.4. Measuring Similarity Between Subreddits over Time with Supervised Dimensionality Reduction

Since UMAP contains parameters that could affect relative distance between subreddits as could downsampling the

data to obtain balanced classes, we estimated the precision of this approach on 2019 data. First, hyperparameter tuning was performed (2700 samples for each subreddit) to find the parameter set that optimized clustering measured through silhouette score using n neighbors (2,10,20,50,100,200), min dist (0.0, 0.1, 0.25, 0.5, 0.8, 0.99) and metric (euclidean, cosine). Second, to tackle the variance caused by subsampling, we measured the pairwise Hausdorff distances between 2019 clusters across 50 runs, each with new random subsampling. Using a distance metric between clusters, rather than their absolute centroid location, allows for avoiding rotation or flipping effects of dimensions. Bootstrapping across runs provides an estimate of the method's precision and also allows us to measure how rare 2020 changes in distances are with respect to a distribution of regular fluctuations for a non-pandemic year (2019). For 2020, we also compute the median distance across 50 bootstrapping samples for our final analysis.

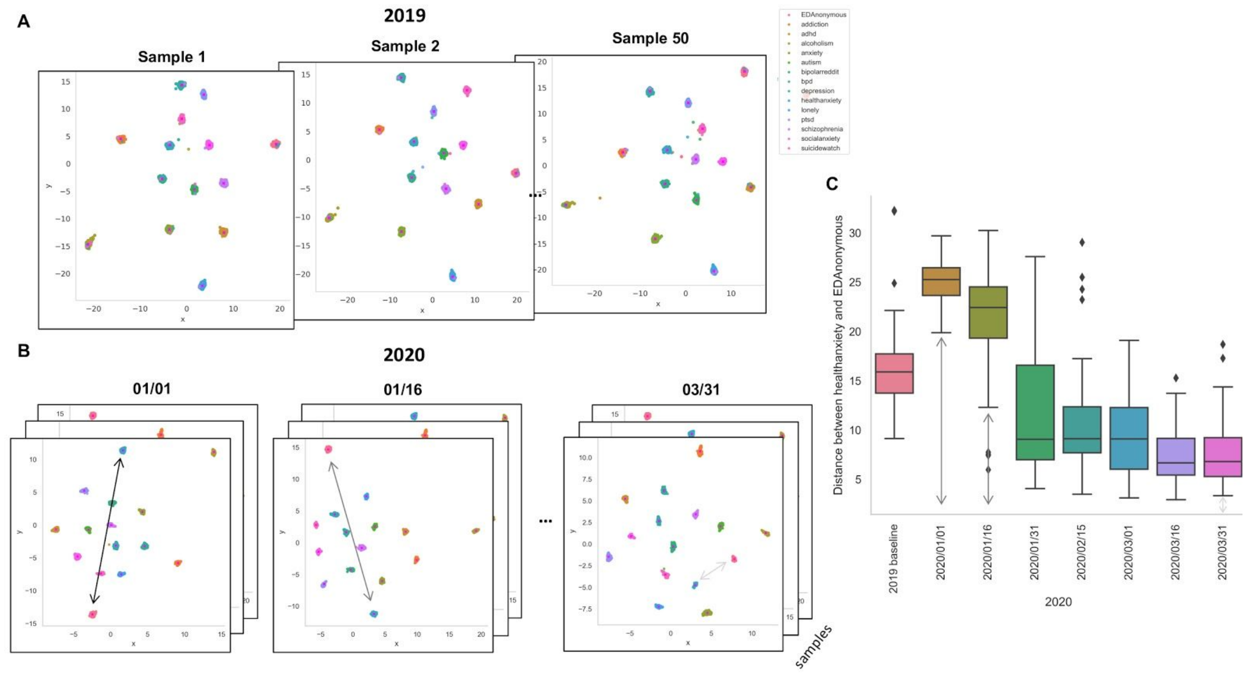

Figure S2. Procedure to establish precision of supervised UMAP for measuring distances between subreddits. A) Normal fluctuations in pairwise distances for a given year when subsampling different random samples resulting in the 2019 distribution as observed in C. **B)** Distances when sampling 15-day time windows during 2020. As an example, arrows represent the distance between r/healthanxiety and r/EDAnonymous. **C)** The distributions built in A and B along with arrows represented in B. This allows us to show which changes in 2020 distances are not likely due to normal fluctuations in the data.

2. Results

2.1. Classification and Interpretability

Subreddit	SGD L1	SGD EN	SVM	ET	XGB
EDAnonymous	0.898	0.898	0.904	0.848	0.887
addiction	0.894	0.877	0.890	0.827	0.885
adhd	0.849	0.855	0.860	0.791	0.831
alcoholism	0.926	0.929	0.936	0.904	0.926
anxiety	0.794	0.704	0.812	0.748	0.805
autism	0.874	0.862	0.879	0.811	0.851
BipolarReddit	0.811	0.789	0.811	0.746	0.787
bpd	0.795	0.784	0.801	0.740	0.777
depression	0.801	0.784	0.795	0.709	0.749
healthanxiety	0.891	0.898	0.906	0.860	0.884
lonely	0.859	0.864	0.863	0.824	0.850
ptsd	0.895	0.887	0.889	0.850	0.883
schizophrenia	0.828	0.811	0.827	0.762	0.800
socialanxiety	0.827	0.839	0.841	0.775	0.833
SuicideWatch	0.830	0.812	0.846	0.780	0.800
**Mean**	**0.851**	**0.840**	**0.857**	**0.798**	**0.837**
**Model complexity No. (%)**	**2503 (48)**	**3332 (64)**	**4316 (83)**	**5190 (100)**	**5190 (100)**

Table S2. Weighted F1 of independent binary classifiers. Model complexity is defined as the amount of non-zero features of the total 5190 features across classifiers (15 × 346). SGD L1: Stochastic gradient descent linear classifier with L1 penalty; SGD EN: Stochastic gradient descent linear classifier with elastic net penalty; SVM: linear support vector machine; ET: extra trees classifier; XGB: extreme gradient boosting classifier.

Subreddit	F1 prepandemic	F1 midpandemic	Change
autism	0.874	0.800	0.074
BipolarReddit	0.811	0.753	0.058
healthanxiety	0.891	0.834	0.057
depression	0.801	0.761	0.04
anxiety	0.794	0.755	0.039
addiction	0.894	0.867	0.027
ptsd	0.895	0.869	0.026
alcoholism	0.926	0.903	0.023
socialanxiety	0.827	0.836	0.009
SuicideWatch	0.830	0.837	0.007
EDAnonymous	0.898	0.891	0.007
bpd	0.795	0.801	0.006
lonely	0.859	0.864	0.005
adhd	0.849	0.852	0.003
schizophrenia	0.828	0.829	0.001
**Mean**	**0.851**	**0.83**	**0.025**

Table S3. Comparing performance of prepandemic baseline model on pre and mid pandemic test sets. Larger differences may indicate dataset shift during the spread of COVID-19.

2.2. Trend Analysis

See Figure S4 for examples of trends and regression. See Figure S5 for 2020 main results and Figures S6 and S7 for comparisons to 2019 and 2018 trends. See Table 2 in the main text for examples of significant trends in Figure S5.

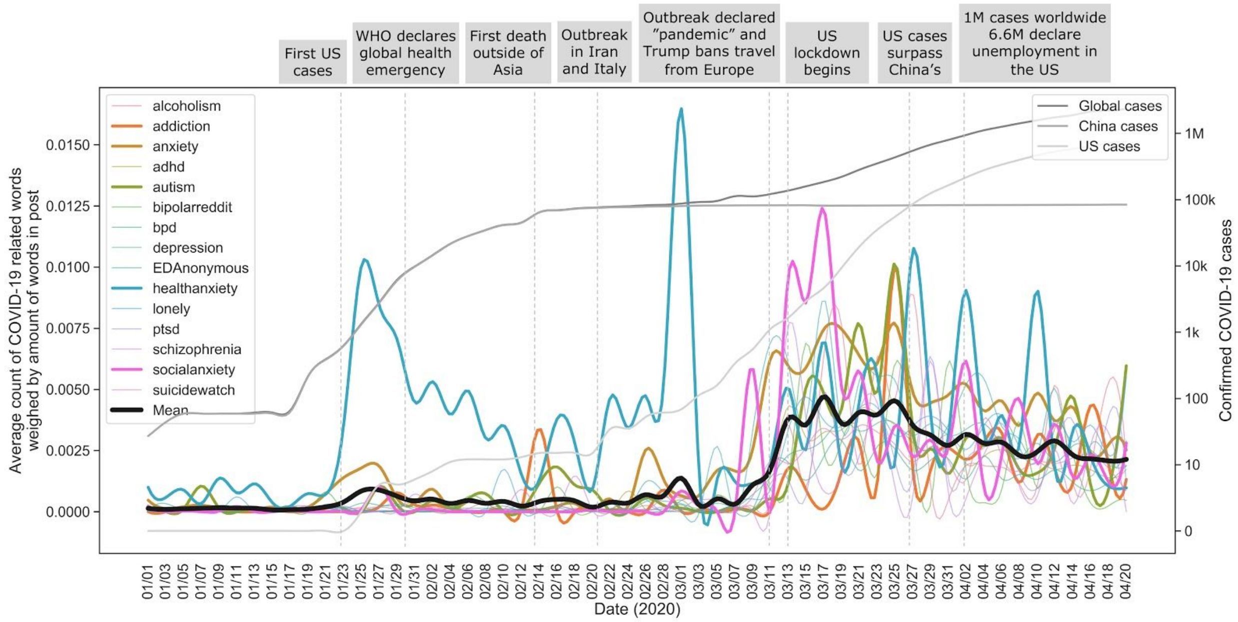

Figure S3. Amount of posts weighed by how much they are about COVID-19.
Timeline landmarks are chosen from NBC News timeline. The acute rise in COVID19 related posts occurs around March 11. The correlation between mean proportion of COVID-19 related posts and global COVID-19 cases is ρ = 0.82 ($P < 0.001$). The health anxiety subreddit has a large increase in COVID-19 related posts almost two months before the general increase. The social anxiety subreddit has an increase during the beginning of the lockdowns in the US.

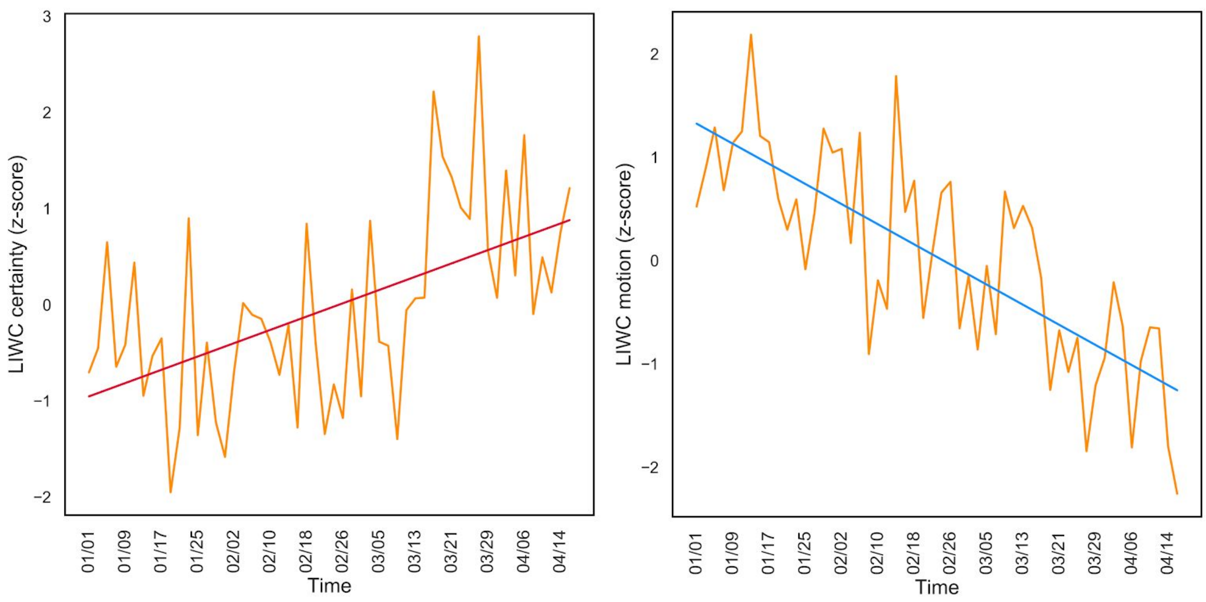

Figure S4. Examples of trends and regression for a single feature and subreddit. Example from r/anxiety of trends and color code for Figure 2 and Figures S5, S6, and S7. The mean value for each time window is computed and then a regression is fit. Positive slopes are in red and negative slopes and in blue.

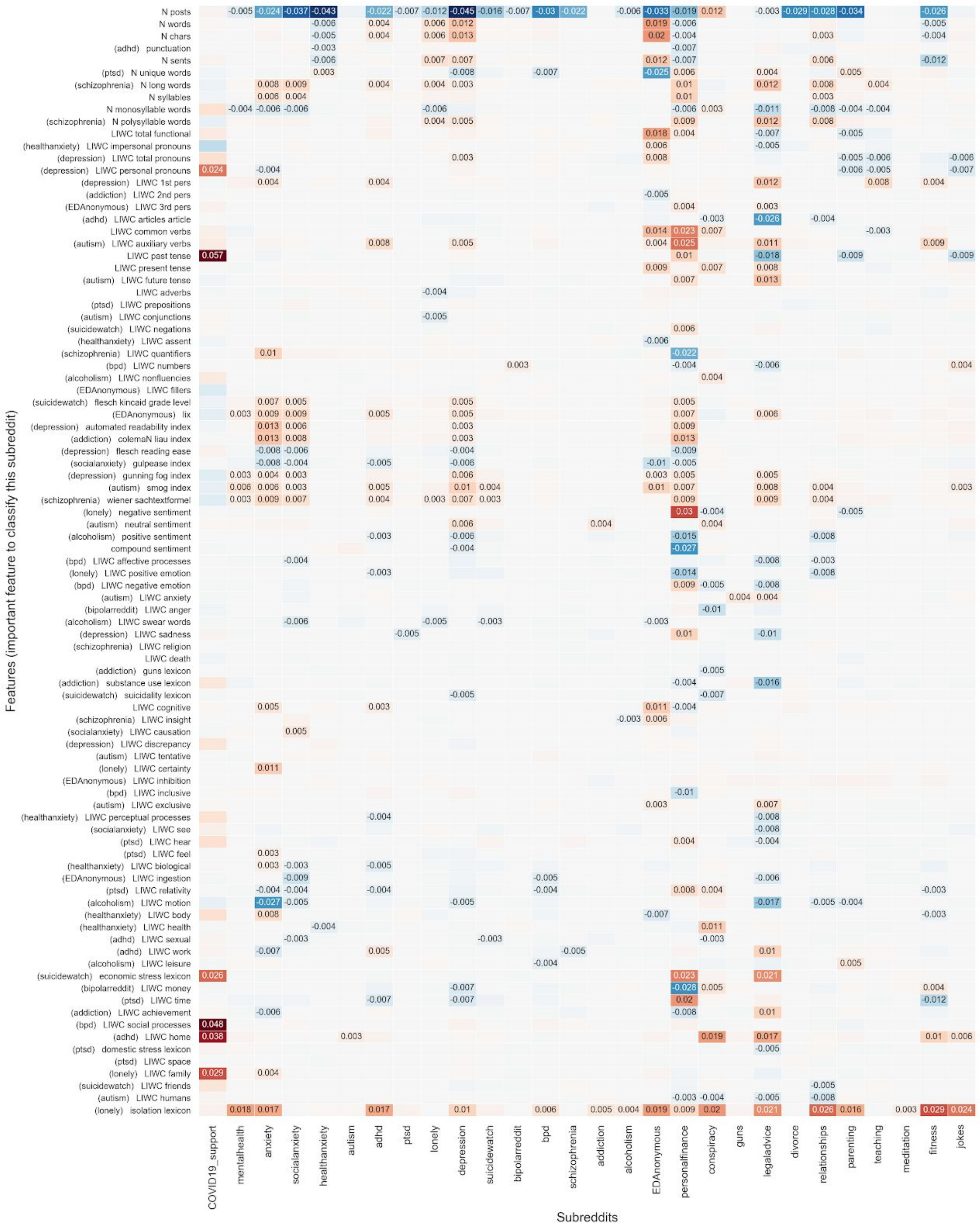

Figure S5. Full results for 2020 trend analysis. Trends of mean feature values across subreddits from 2020-03-11 to 2020-03-20. We concatenated for which binary classifier they have the highest coefficient and thus are important to classify (only using upper half of coefficient values, which is why not all features are linked to a subreddit). Benjamini-Hochberg procedure was applied for multiple comparison (uncorrected alpha = 0.05) and non-significant results are masked.

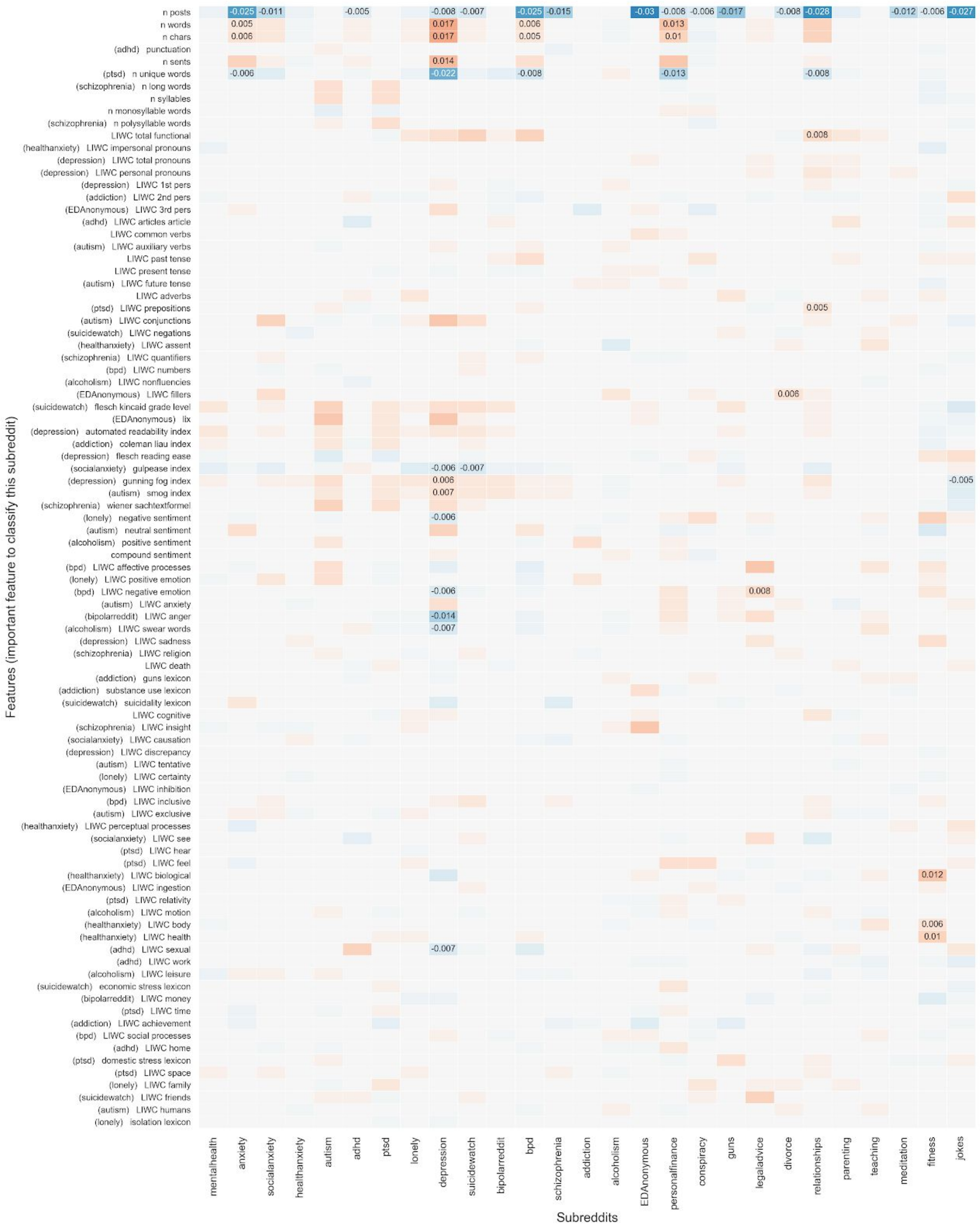

Figure S6. 2019 trend analysis. Trends of mean feature values across subreddits from 2019-03-11 to 2019-03-20. See Figure S5 for more details.

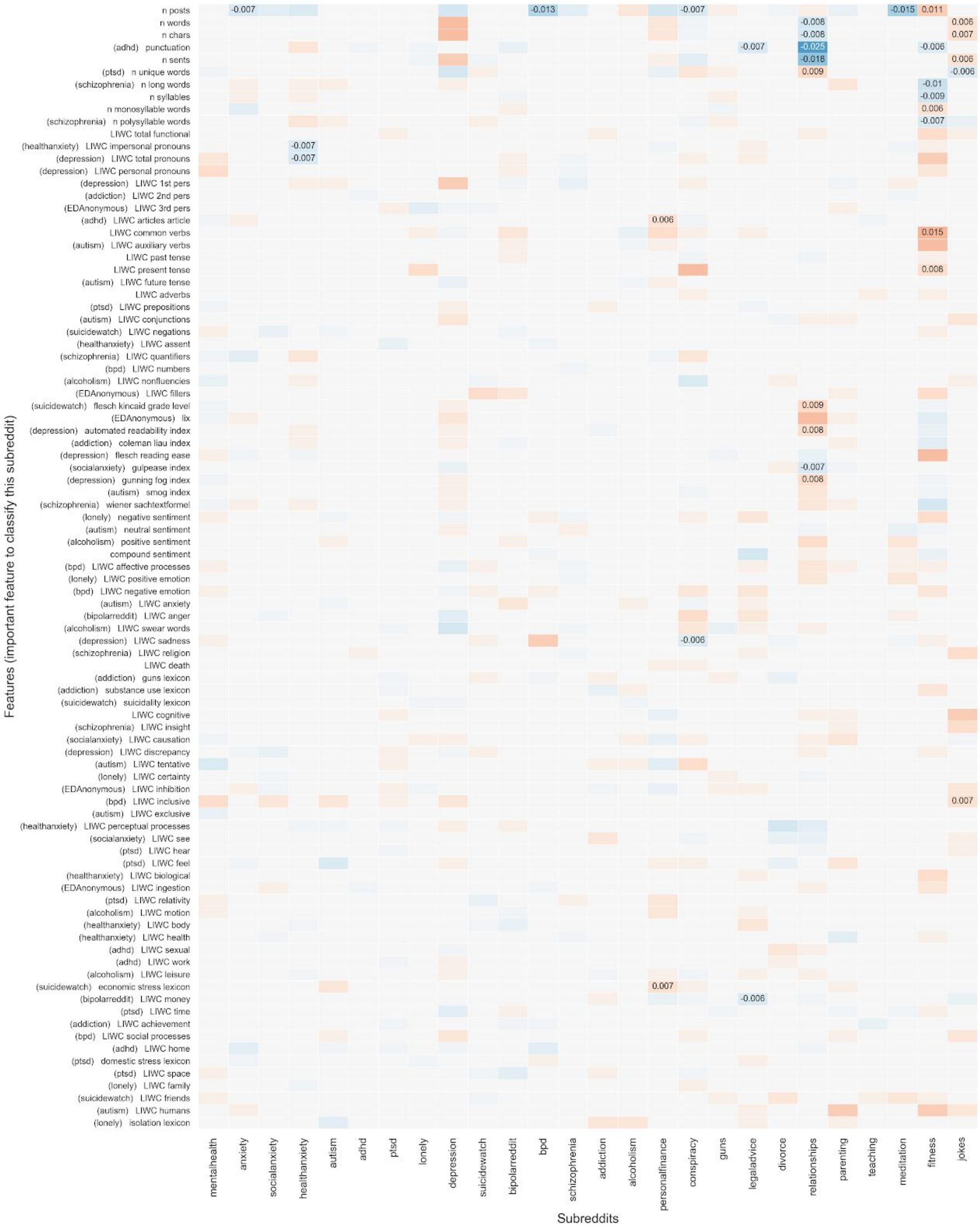

Figure 7. 2018 trend analysis. Trends of mean feature values across subreddits from 2018-03-11 to 2019-03-20. See Figure S5 for more details.

2.3. Unsupervised clustering

Cluster Annotation	Distinguishing Features, Cluster of PrePandemic Posts		Distinguishing Features, Cluster of PrePandemic Posts	
	N	Example Features	N	Example Features
Alcohol	31	Substance Use Lexicon total, LIWC features {'ingestion', 'inhibition', 'leisure'}, TF-IDF features {'addict', 'alcohol', 'beer', 'day', 'drank', 'drink', 'drunk', 'everi', 'last', 'month', 'night', 'problem', 'quit', 'sober', 'stop', 'week', 'weekend', 'withdraw'}	20	Substance Use Lexicon total, LIWC features {'ingestion', 'leisure'}, TF-IDF features {'alcohol', 'beer', 'day', 'drink', 'drunk', 'everi', 'night', 'quit', 'sleep', 'sober', 'stop', 'withdraw'}
Alcohol Longer Posts	187	Number of characters, long words, sentences, unique words	128	Number of characters, long words, sentences, unique words, Substance Use Lexicon total, LIWC features {'ingestion', 'inhibition', 'leisure', 'quantifiers', 'social_processes'}, TF-IDF features {'addict', 'alcohol', 'beer', 'drank', 'drink', 'drunk', 'problem', 'quit', 'sober', 'stop'}
Eating	18	LIWC features {'biological', 'ingestion'}, TF-IDF features {'ate', 'bing', 'bodi', 'calori', 'eat', 'ed', 'fat', 'food', 'gain', 'lose', 'meal', 'purg', 'restrict', 'weight'}	15	LIWC features {'biological', 'ingestion'}, TF-IDF features {'bing', 'calori', 'eat', 'ed', 'fat', 'food', 'gain', 'lose', 'purg', 'restrict', 'weight'}
Eating Longer Posts	101	Numbers of characters, words, and sentences, LIWC features {'body', 'ingestion', 'leisure', 'motion'}, TF-IDF features {'amount', 'ate', 'bing', 'bodi', 'calori', 'control', 'diet','eat', 'ed', 'exercis', 'fat', 'food', 'gain', 'pound', 'purg', 'restrict', 'weight' }	101	Numbers of characters, words, and sentences, LIWC features {'body', 'ingestion', 'leisure', 'motion'}, TF-IDF features {'bing', 'bodi', 'calori', 'control', 'diet', 'disord', 'eat', 'ed', 'enough', 'fat', 'food', 'gain', 'lose', 'meal', 'purg', 'restrict', 'weight' }
Entertain-ment			8	Positive Sentiment, Compound Sentiment, LIWC features {'leisure', 'positive_emotion'}, TF-IDF features {'game', 'play', 'video', 'watch'}
General Mental Health	14	LIWC features {'health'}, TF-IDF features {'health', 'ill', 'mental', 'mental health', 'mental ill'}		
Health Anxiety	90	LIWC features {'anxiety', 'biological', 'body', 'health'}, TF-IDF features {''anxieti', 'arm', 'attack', 'back', 'blood', 'bodi', 'brain', 'breath', 'cancer', 'caus', 'check','chest', 'doctor','fear', 'freak', 'googl', 'head', 'headach', 'health', 'health anxieti', 'heart', 'pain', 'panic', 'scare', 'side', 'symptom', 'test', 'weird', 'worri'}	81	LIWC features {'anxiety', 'biological', 'body', 'health'}, TF-IDF features {''anxieti', 'attack', 'back', 'blood', 'bodi', 'breath', 'cancer', 'caus', 'check','chest', 'doctor','fear', 'freak', 'googl', 'head', 'headach', 'health', 'health anxieti', 'heart', 'pain', 'panic', 'scare', 'side', 'symptom', 'test', 'weird', 'worri'}
Loneliness	28	Isolation Lexicon total, LIWC features {'friends', 'sadness', 'social_processes'}, TF-IDF features {'alon', 'feel', 'feel like',	6	Flesch Reading Ease, Gulpease Index, Negative Sentiment, Positive Sentiment, Isolation Lexicon Total, TF-IDF features

		'friend', 'hang', 'like', 'lone', 'love', 'make', 'one', 'peopl', 'sad', 'someon', 'talk', 'want'}		{‘lone’}
Long Posts	440	Number of: characters, long words, monosyllabic words, polysyllabic words, sentences, syllables, unique words	477	Number of: characters, long words, monosyllabic words, polysyllabic words, sentences, syllables, unique words
Marijuana	10	TF-IDF features {’addict’, ’drug’, ’high’, ’quit’, ’smoke’, ’smoke weed’, ’weed’}	20	TF-IDF features {’addict’, ’drug’, ’high’, ’quit’, ’smoke’, ’smoke weed’, ’weed’}
Medication	43	LIWC features {'biological', 'causation', 'health', 'insight', 'numbers', 'time', 'work'}, TF-IDF features {'day', 'doctor', 'dose', 'effect', 'experi', 'med', 'medic', 'mg', 'prescrib', 'side effect', 'start', 'take', 'took', 'week', 'work'}	17	LIWC features {'causation', 'health'}, TF-IDF features {'effect', 'med', 'medic', 'side', 'side effect','take'}
Panic Attacks	9	Negative sentiment, LIWC features {affective processes, anger, anxiety, negative emotion}, TF-IDF features {’anxieti’, ’attack’, ’panic’, ’panic attack’}	13	Negative sentiment, LIWC features {affective processes, anger, anxiety, body, negative emotion}, TF-IDF features {’anxieti’, ’attack’, ‘breath’, ’panic’, ’panic attack’}
Pleading	12	TF-IDF features {’help’, ’please’, ’cant’, ’dont’, ’dont know’, ’know’,’im’, ’ive’}, Flesch Reading Ease, Gulpease Index	12	TF-IDF features {‘anymor’,’cant’, ’dont’, ’dont know’, ’know’,’im’, ’ive’, ‘want’}, Flesch Reading Ease, Gulpease Index, Suicidality Lexicon Total, LIWC features {‘death’}
Resources	17	TF-IDF features {'com', 'https', 'https www', 'share', 'www'}	16	TF-IDF {'com', 'help', 'https', 'https www', 'www'}
Resources	12	TF-IDF features {’com’, ’https’, ’https www’, ’www’}	12	TF-IDF features {'com', 'https', 'https www', 'www'}
Resources			7	TF-IDF features {’https’, ’https www’, ’www’}
Seeking Advice	13	Neutral Sentiment, Compound Sentiment, TF-IDF features {'advic', 'ani', 'ani advic', 'ani tip', 'anyon', 'anyon ani', 'appreci', 'doe', 'doe anyon', 'help', 'tip'}		
Seeking Normali-zation	7	Coleman Liau index, Neutral Sentiment, TF-IDF features {'anyon', 'anyon els', 'doe', 'doe anyon', 'els'}	8	Coleman Liau index, Neutral Sentiment, TF-IDF features {'anyon', 'anyon els', 'doe', 'doe anyon', 'els'}, Automated Readability Index
Self Harm			11	Negative Sentiment, Suicidality Lexicon Total, LIWC features {'affective_processes', 'death', 'humans', 'negative_emotion'}, TF-IDF features {'harm', 'self', 'self harm', 'suicid'}
Social Anxiety	13	TF-IDF features {’anxieti’, ’awkward’, ’peopl’, ’social’, ’social anxieti’, ’talk’}	15	LIWC features {‘social_porcesses’}, TF-IDF features {’anxieti’, ‘anxious', ’awkward’, ’peopl’, ’social’, ’social anxieti’, ’talk’}
Suicidality	23	Sentiment Negative, Suicidality lexicon total, LIWC features {'anger', 'death', 'negative_emotion', 'sadness', 'sexual', 'swear_words'}, TF-IDF features	349	Number of characters, words, sentences; Totals for the COVID-19, Economic Stress, Isolation, Domestic Stress and Suicidality Lexicons; LIWC features {‘anger’, 'anxiety',

		{'anymor', 'die', 'end', 'fuck', 'hate', 'kill', 'life', 'live', 'noth', 'shit', 'suicid', 'want', 'want die'}		‘death’,'family', 'friends', 'future_tense', 'home', 'money', 'motion', 'negative_emotion', 'sadness', 'sexual', ‘social_processes’, 'swear_words', ‘work’}, TF-IDF features {‘abus’, 'anymor', ‘college’, 'die', 'end', 'fuck', 'hate', 'kill', 'life', 'live', 'noth', 'shit', 'suicid', 'want'}
Unspecified	9	Automated readability index, Coleman Liau index, Flesch Kincaid Grade Level, Gunning Fog Index, LIX, Smog Index, Wiener Sachtextformel, Neutral Sentiment, Compound Sentiment	22	Automated readability index, Coleman Liau index, Flesch Kincaid Grade Level, Gunning Fog Index, LIX, Smog Index, Wiener Sachtextformel, Neutral Sentiment, Compound Sentiment, COVID-19 Lexicon Total, TF-IDF features {‘advic’, ‘ani’, ‘diagnos’, ‘diagnosi’, ‘experi’, ‘med’, ‘medic’, ‘tip’}
Unspecified 2	350	Automated readability index, Flesch-Kincaid grade level, Gunning fog index, Smog index, Numbers of words, syllables, and sentences, LIWC features {’causation’, ’family’, ’home’, ’insight’, ’leisure’, ’money’, ’religion’, ’sexual’}		

Table S4. Cluster annotations. Cluster annotations were assigned based on a review of the features found to distinguish each cluster, using Wilcoxon rank-sum tests with Bonferroni correction. Representative significantly cluster-associated features informing the annotation are shown, along with the total number of significant features per cluster. Clustering was performed separately with k=20 on the prepandemic posts dataset from 2019 and the midpandemic posts dataset from 2020. The majority of clusters were approximately replicated between the two time periods. Each pair of clusters (one from the prepandemic data and one from the midpandemic data) assigned the same annotation are shown side-by-side to illustrate the overlap of their predictive features. A few clusters were detected only in the prepandemic dataset (e.g., General Mental Health, Seeking Advice) and a few were only detected in the midpandemic dataset (e.g., Self Harm, Entertainment). Two “Resources” clusters were detected in the prepandemic dataset, and three were detected in the midpandemic dataset. The characteristic features were not meaningfully distinct for any among these “Resources” clusters and these clusters partitioned an island of posts visible in UMAP space, so all “Resources” clusters detected in a given dataset were collapsed into a single cluster.

	Attributes for Cluster of PrePandemic Posts		
**Cluster Annotation**	**Diversity (Shannon Index)**	**Size (Number of Posts)**	**Mean Post Length**
Eating Longer Posts	1.09	218	364.08
Health Anxiety	1.30	1105	193.50
Social Anxiety	1.37	421	107.62
Eating	1.42	1009	108.04
Alcohol	1.67	1067	155.89
Alcohol Longer Posts	2.08	238	553.95
Marijuana	2.10	246	161.64
Suicidality	2.63	1438	112.94
Loneliness	2.67	1620	121.41
Panic Attacks	2.76	413	150.87
Medication	2.87	412	176.71
Resources	3.56	490	118.88
General Mental Health	3.59	416	135.82
Seeking Advice	3.61	618	92.71
Long Posts	3.67	534	885.73
Unspecified 2	3.71	4272	318.10
Seeking Normalization	3.72	739	71.50
Pleading	3.74	915	81.22
Unspecified	3.78	6329	94.32

Table S5. Cluster attributes on prepandemic posts. Cluster attributes, including total cluster size in number of posts, mean post length, and diversity are shown for each cluster from the prepandemic post dataset. Diversity was computed with the scikit-bio package of alpha diversity measures using the Shannon Diversity Index based on the representation within the cluster of posts across the 15 subreddits included in the analysis. Higher values indicate greater diversity. Clusters are sorted in ascending order according to their diversity.

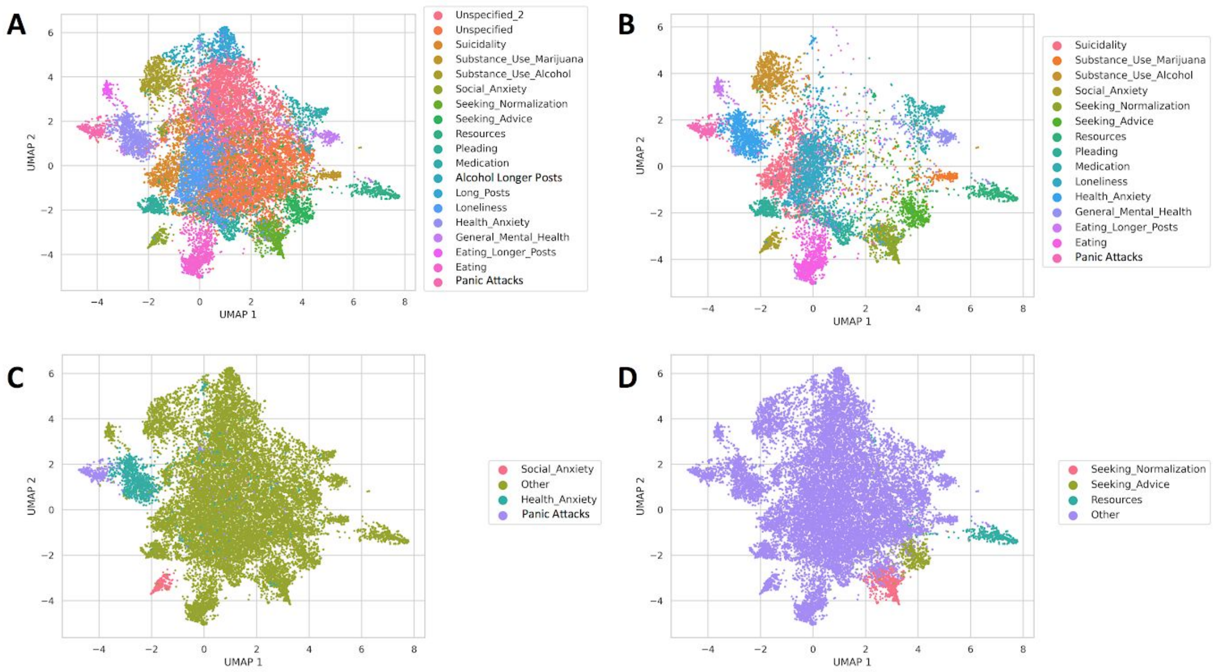

Figure S8. Unsupervised clustering of posts made in 2019 to 15 mental health subreddits. Values of k from 5 to 50 were tested in increments of five and the quality of the resulting graph partitions were evaluated with a modularity metric. Modularity scores rose to a plateau of approximately 0.65 for k ≥ 10 and k=20 was selected because it yielded sizable clusters with more interpretable cluster-distinguishing features than k=10 or 15. **A)** UMAP with all clusters displayed with equal point translucency. **B)** UMAP with the two largest, and the two containing the longest posts, removed to highlight proximities among the remaining clusters. **C)** Highlighting the proximity of anxiety-related clusters. D) Highlighting the proximity of clusters related to resource provision and advice seeking.

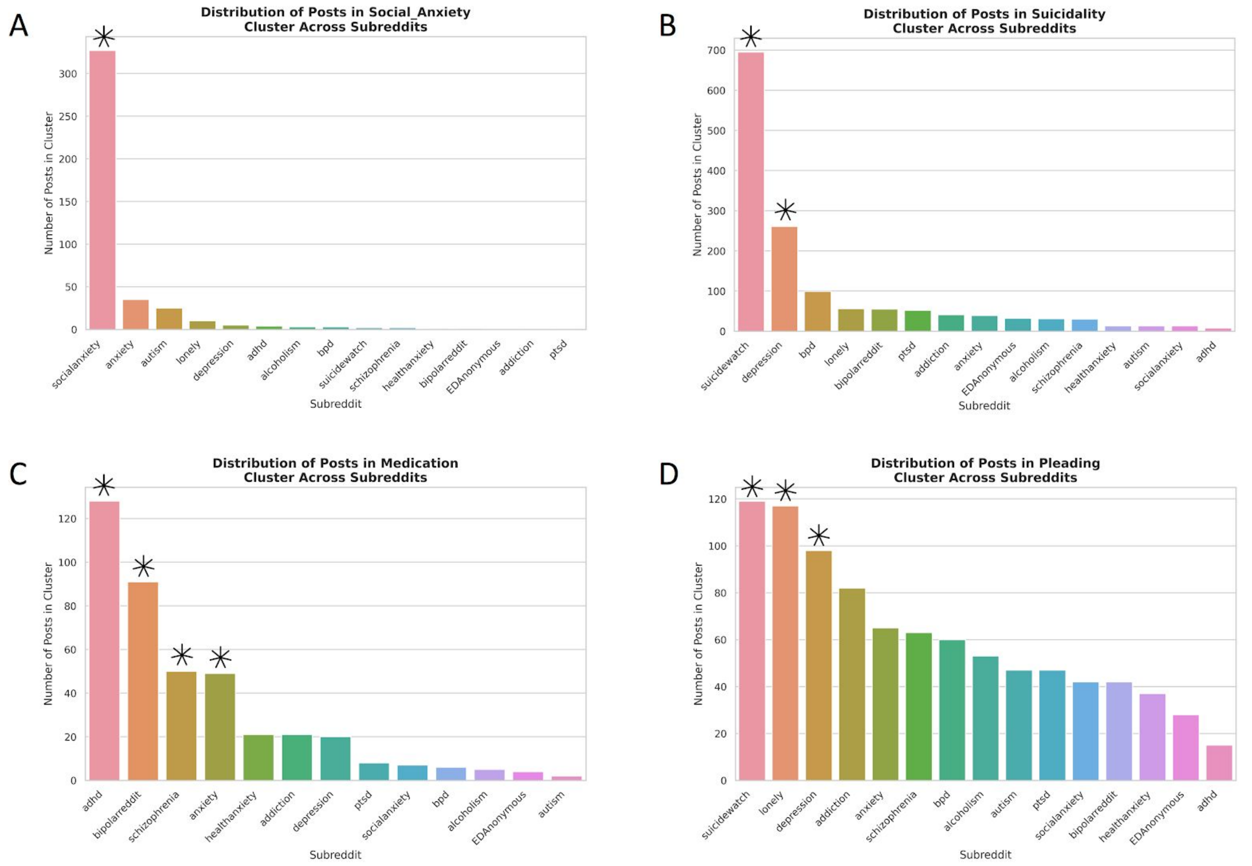

Figure S9. Representation of posts from each subreddit within a given cluster for the 2019 prepandemic dataset.

A) the Social Anxiety cluster, **B)** the Suicidality cluster, **C)** the Medication cluster, **D)** and the Pleading cluster. Columns are marked with stars for subreddits on which posts from the given cluster were significantly enriched. See Figure 3B for the full set of cluster enrichments on the analysed subreddits.

2.4. Topic Modeling with Latent Dirichlet Allocation (LDA)

We analyzed whether the subreddits that most increased in their Health Anxiety topic correlated with the ones that most increased in negative semantic change as measured by the trend analysis, but this was not significant (ρ = -0.046, P = 0.819).

Topic	Words
Autism/ADHD + School/Work	autism, work, help, adhd, school, people, autist, https, time, need
Alcohol/Addiction	year, addic, time, go, know, month, help, drink, want, work
Sleep Issues	go, sleep, night, fuck, time, hour, tell, come, say, know
Alcohol/Eating Disorders	drink, eat, weight, alcohol, food, feel, bing, lose, week, know
Social Interaction	friend, peopl, talk, feel, know, social, want, think, time, school
Schizophrenia	think, thing, feel, know, experi, hear, peopl, voic, thought, schizophrenia
Medical/Medication	medic, take, depress, med, feel, bipolar, doctor, help, start, work
Health Anxiety	anxieti, feel, go, attack, panic, pain, hert, start, doctor, week
Mental Health Help	help, know, tell, mental, year, say, want, thing, diagnos, think
Life	feel, want, life, know, think, love, time, thing, go, peopl

Table S6: PrePandemic Topic Model. Topics extracted from LDA on prepandemic mental health subreddits.

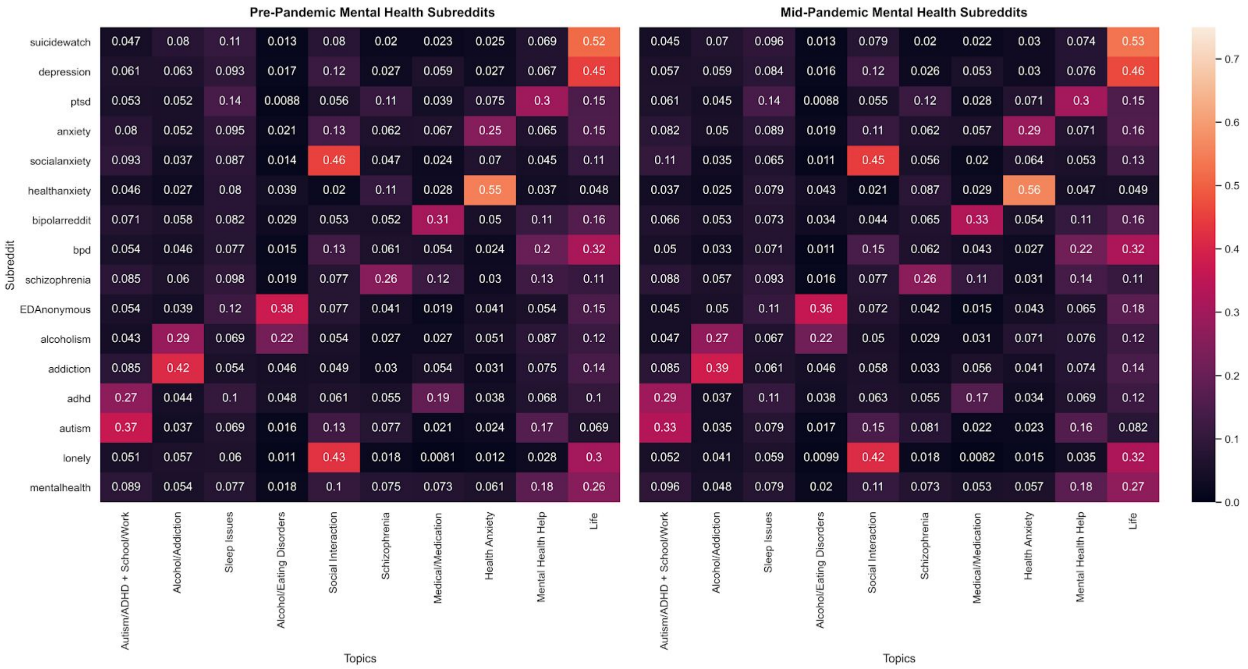

Figure S10: Prepandemic LDA model distribution over mental health subreddits. Distribution of prepandemic LDA topics for posts in mental health subreddits prepandemic (left) and midpandemic (right). This highlights that the distributions of these particular topics within a single subreddit largely did not change between pre and midpandemic timeframes, except for an increase in the topics "Health Anxiety" and "Life" and a decrease in the "Alcohol/Addiction" topic.

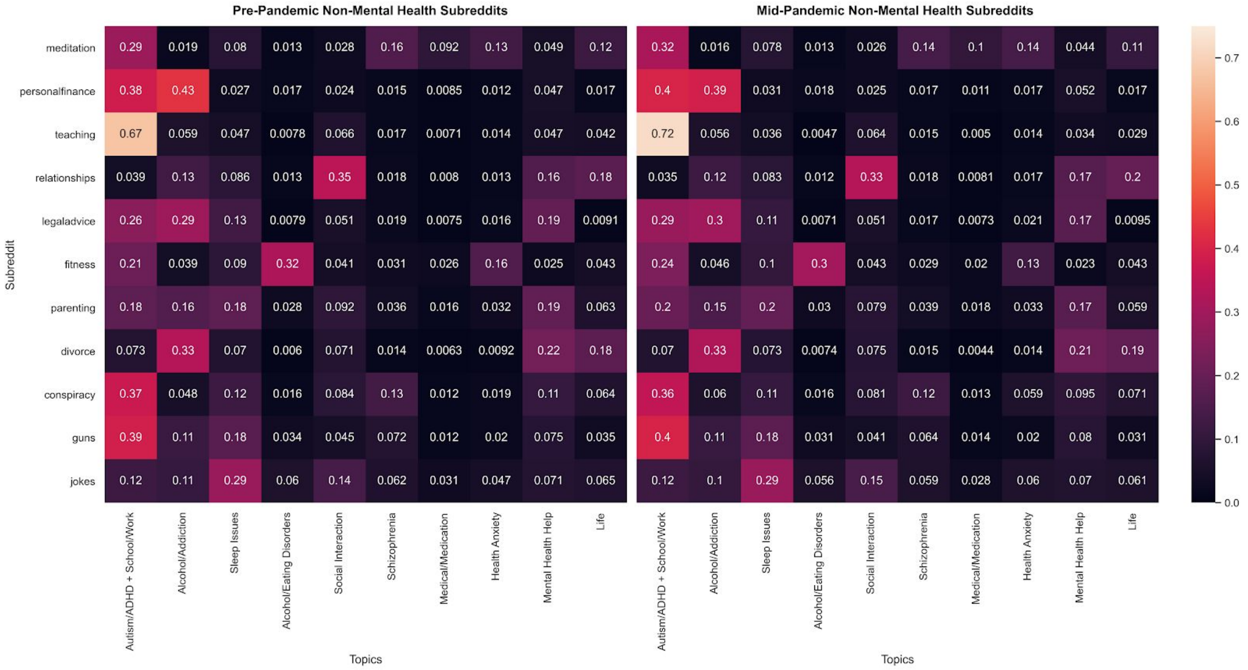

Figure S11: Prepandemic LDA model over non-mental health subreddits. Distribution of prepandemic LDA topics for posts in non-mental health subreddits prepandemic (left) and midpandemic (right). As with the mental health subreddits, distributions of these particular topics within a single subreddit largely did not change between pre

and midpandemic timeframes, except for an increase in the topics “Health Anxiety” and “Life” and a decrease in the “Alcohol/Addiction” topic.

2.5. Measuring Similarity Between Subreddits over Time with Supervised Dimensionality Reduction

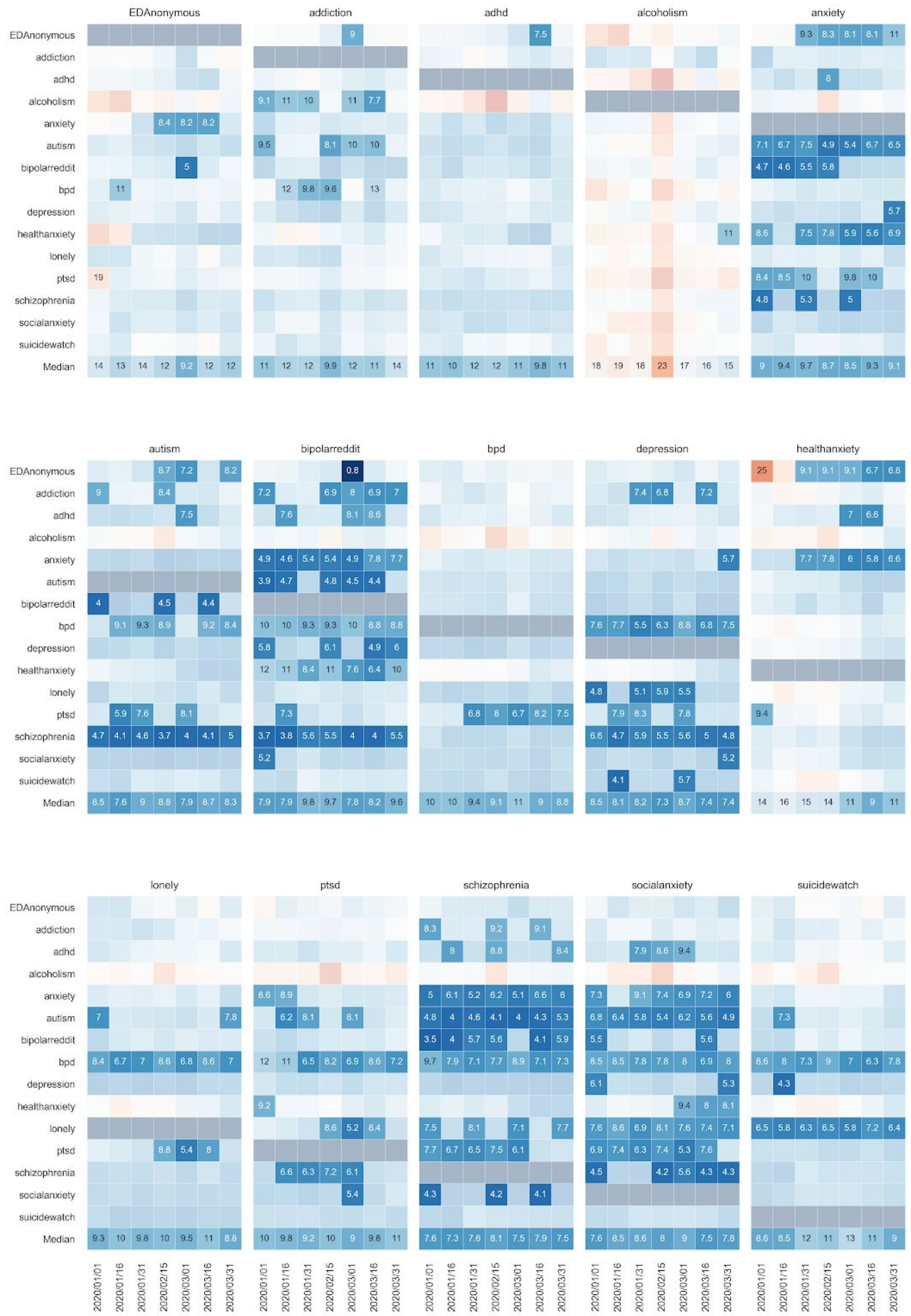

Figure S12. Pairwise changes in distance between subreddits throughout pandemic. Total pairwise distance between subreddits for each time window displaying only extreme values with regards to normal 2019 fluctuations

(top and bottom 5th percentiles), which indicates they are less likely to be part of regular fluctuations in distance (see Supplement 1.4 for a precision analysis of this approach).

References

1. Hutto CJ, Gilbert E. Vader: A parsimonious rule-based model for sentiment analysis of social media text. Proceedings of the 8th International AAAI Conference on Weblogs and Social Media 2014. Available from: https://www.aaai.org/ocs/index.php/ICWSM/ICWSM14/paper/viewPaper/8109

2. DeWilde B. textacy Documentation. 2017. Available from: https://buildmedia.readthedocs.org/media/pdf/textacy/latest/textacy.pdf

3. Pennebaker JW, Booth RJ, Boyd RL, Francis ME. LIWC 2015 Operator's Manual. Austin, TX: Pennebaker Conglomerates Inc; 2015.
